# Development of antibody drug conjugates targeting epithelial membrane protein 2-highly expressed lung cancer

**DOI:** 10.1038/s41419-025-08125-7

**Published:** 2025-10-31

**Authors:** Mengge Zheng, Su Hang, Changyong Hu, Jianxia Chen, Xinshuai Wang, Xiangyang Wu, Junfang Xu, Li Chen, Yajuan Cao, Jiani Gao, Likun Hou, Chunyan Wu, Xun Meng, Chang Chen, Haipeng Liu

**Affiliations:** 1 Central Laboratory, Shanghai Pulmonary Hospital, Tongji University School of Medicine, Shanghai, China; 2 Department of Thoracic Surgery, Shanghai Pulmonary Hospital, Tongji University School of Medicine, Shanghai, China; 3 Institute of Metabolism & Integrative Biology (IMIB), Fudan University, Shanghai, China; 4 Shanghai Key Laboratory of Tuberculosis, Shanghai Pulmonary Hospital, Tongji University School of Medicine, Shanghai, China; 5 Department of Integrated Traditional Chinese and Western Medicine, Shanghai Pulmonary Hospital, Tongji University School of Medicine, Shanghai, China; 6 Department of Pathology, Shanghai Pulmonary Hospital, Tongji University School of Medicine, Shanghai, China; 7 Abmart, Shanghai, China

**Keywords:** Targeted therapies, Non-small-cell lung cancer

## Abstract

Lung cancer is one of the most devastating types of cancer, and the treatment of lung cancer has been facing great challenges. Antibody-drug conjugates (ADCs), a new type of targeted therapy, have been widely used in cancer therapy and have opened up a new perspective for the treatment of lung cancer. Here, using a tissue-microarray-based antibody library screening, we identified the FK002 antibody that specifically binds to the tumor cell membrane across various tumor types. We determined that FK002 targets epithelial membrane protein 2 (EMP2), a member of the tetraspanin superfamily of proteins. Subsequently, we developed the EMP2-directed ADC, FK002-exatecan, with a potent DNA topoisomerase I inhibitor (exatecan). In-depth in vitro and in vivo experiments have shown the efficacy and specificity of the EMP2-directed ADC. We validated that the FK002-exatecan ADC effectively eradicated tumors in various lung cancer cell lines and xenograft mouse models, including patient-derived xenograft (PDX) and patient-derived tumor-like cell cluster (PTC) models, as well as xenograft tumors. Mechanistically, FK002-exatecan specifically bound to EMP2 and was internalized into tumor cells, followed by intracellular trafficking to the lysosome and exatecan release, which induced cell cycle arrest and apoptosis. This study identifies EMP2 as a novel molecular target for lung cancer therapy and establishes a foundation for developing ADCs that selectively eradicate lung cancer cells.

## Introduction

Lung cancer remains one of the leading causes of cancer-related death worldwide. Non-small cell lung cancer (NSCLC) stands out as the predominant histological subtype, boasting a modest 5-year survival rate of around 15% across all stages [1]. Although the advent of molecular targeted therapy and immunotherapy has significantly improved the prognosis of NSCLC patients, the overall prognosis of NSCLC remains dismal, with a 5-year survival rate of 16% as reported [2, 3]. Additionally, the efficacy of immunotherapy is largely limited to a low response rate (20–30%) [4]. This illustrates the imperative for more effective therapies to improve patient outcomes.

Antibody-drug conjugates (ADCs) are one of the fastest-growing anticancer drugs. They are composed of three core components: an antibody that recognizes a tumor-associated antigen, a chemical linker, and a cytotoxic payload [5]. It is a therapeutic outgrowth of the “magic bullet” concept championed by Paul Ehrlich more than a century ago [6], designed to meet oncologists’ need for weapons that efficiently target tumor cells with high precision and specificity. ADCs provide a means of increasing the efficacy of chemotherapy by increasing the accumulation of cytotoxic drugs within tumor cells while reducing the nonspecific cytotoxic effects of systemic drug administration [7, 8]. It combines the advantages of highly specific targeting ability and potent killing effect to achieve accurate and efficient elimination of cancer cells, which has become one of the hotspots for the research and development of anticancer drugs. Currently, the FDA has approved 16 ADCs for exclusively oncology indications. A study of the antibody-drug conjugate target landscape provides a data-driven prioritization of clinically available ADCs directed against 59 unique targets across 60 tumor (sub)types [9]. In recent years, the approval of several new ADCs for solid tumor treatment has been a promising development. Trastuzumab deruxtecan, an anti-HER2 ADC, has demonstrated significant efficacy in HER2-overexpressing lung cancer, breast cancer, stomach cancer, and colorectal cancer [10–13], indicating a wide-ranging potential for ADCs in solid tumor therapy.

Increasing evidence highlights epithelial membrane protein 2 (EMP2) as a promising therapeutic target in various human cancers [14–18]. EMP2 belongs to the peripheral myelin protein 22kD (PMP22) gene family, which consists of at least seven members: PMP22, EMP1, EMP2, EMP3, PERP, brain cell membrane protein 1, and MP20 [19, 20]. It is predicted to contain two extracellular domains, a small cytoplasmic tail, and three putative N-linked glycosylation sites [16]. EMP2 plays a vital regulatory role in blastocyst implantation, cell division, adhesion, and migration [21–25]. Alterations in EMP2 have been observed across different cancer types, suggesting its significance in tumorigenesis [14, 15, 26]. Notably, a study by Wang et al. revealed that EMP2 exhibits organ-specific functions in cancer pathogenesis, acting as an oncogene in hormone-related cancers such as human endometrial cancer and ovarian cancer while functioning as a tumor suppressor in urothelial cancer [16]. Mechanistically, EMP2’s regulatory role in activating FAK and Src kinases drives endometrial tumor formation and enhances glioblastoma (GBM) growth [18, 27]. Initially, Morales et al. established the link between EMP2 and vascular endothelial growth factors (VEGF) in ARPE-19 cells, with Gordon et al. confirming EMP2’s influence on angiogenesis in endometrial cancer through VEGF induction [28, 29]. Notably, tissue microarray (TMA) demonstrated a significant incremental rise in EMP2 expression levels from benign to malignant endometrial cancer [26]. Studies have indicated heightened EMP2 protein levels in 63% of invasive breast cancer and 73% of triple-negative tumors [14]. Furthermore, Qin et al. observed elevated EMP2 expression in glioblastoma tissue compared to healthy brain tissue with minimal or no EMP2 expression [18]. This all indicates that EMP2 may be a potential ADC therapeutic target in cancers exhibiting elevated EMP2 levels.

Mitsui et al. first synthesized a water-soluble and non-pro-drug analog of camptothecin, DX-8951f, which exhibited potent antitumor activity against human tumors in vitro and in vivo [30]. Exatecan (DX-8951) is a synthetic analog of the DNA topoisomerase I inhibitor camptothecin, which was synthesized to increase its water solubility and antitumor activity while reducing toxicity. In this investigation, we employed tissue-microarray screening based on a proteome-scale antibody array platform for the Proteome Epitope Tag Antibody Library (PETAL) to pinpoint antibodies with high specificity for solid tumors [31]. Through target identification, we determined that FK002Ab specifically targeted the human EMP2 protein on the surface of tumor cells. And based on this, we constructed a novel ADC specifically targeting to kill EMP2-highly expressed lung cancer. We aimed to evaluate EMP2 as a viable target for solid tumors, culminating in the development of the antibody-drug conjugates FK002-exatecan designed to target and eliminate tumors with precision.

## Results

### An atlas of lung cancer cell surface–binding monoclonal antibodies (mAbs) by PETAL

PETAL contains 62,208 monoclonal antibodies (mAbs) against 15,199 peptides from diverse proteomes [32]. We used the PETAL, an existing library, to construct a lung cancer cell surface antibody-target atlas for ADCs development. Our antibody screening process is divided into two stages. The first stage involved screening antibodies based on their expression characteristics (cellular localization and intensity) in lung cancer cell lines via flow cytometry assays. Fluorescently labeled live lung cancer mixed cell lines, including human lung squamous carcinoma cell lines NCI-H226, NCI-H520, SK-MES-1, and NCI-H2170, and human small cell lung cancer cell lines NCI-H69 and NCI-H526, were used to screen the PETAL library. At this screening stage, we defined a mean fluorescence intensity (MFI) >1 as the threshold for potential positivity in flow cytometry results. Using this criterion, we identified 940 cell-binding monoclonal antibodies (mAbs) that showed positive binding signals in our flow cytometry assays (Supplementary Table 1). Then, 85 membrane protein-labeled mAbs were screened again using the above single-cell lines to assess the expression levels of their targets in lung cancer cells (Supplementary Table 2).

In the second stage, screening was performed using human lung cancer samples. The 85 membrane protein-labeled mAbs were further evaluated across several pathological subtypes of lung cancer, including lung adenocarcinoma (*n* = 30), squamous cell lung carcinoma (*n* = 25), small cell lung cancer (*n* = 25), and paired normal adjacent tissues (NATs) by immunohistochemistry (IHC) staining. Finally, 12 mAbs showed elevated lung cancer expression relative to NATs by IHC; the remaining 73 mAbs were excluded either because of low expression positivity (≤5%) or poor tumor cell expression specificity.

**Fig. 1:**
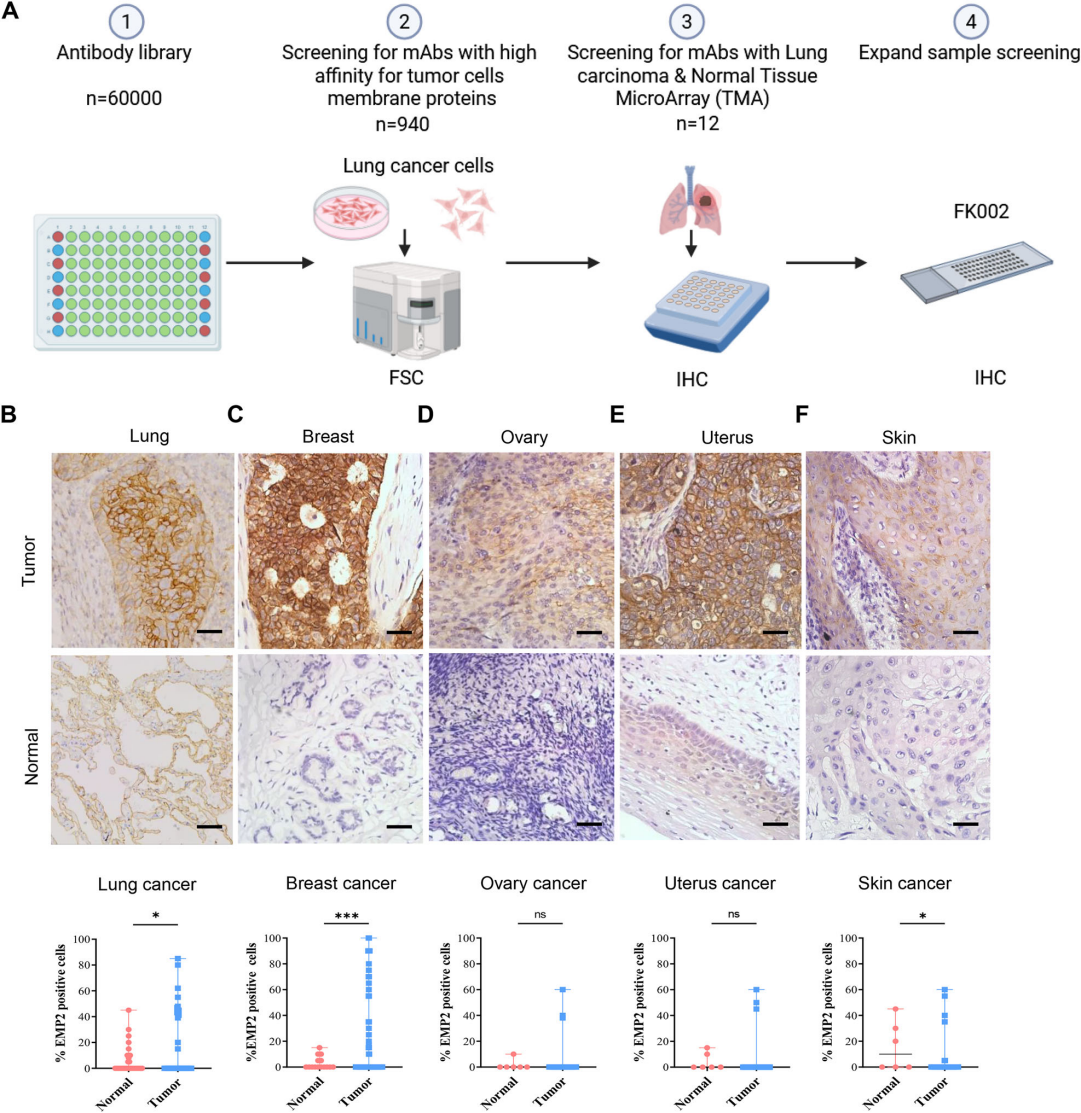
Target antigen of FK002 is overexpressed in various human tumors. **A** Schematic for constructing a cell surface mAb-target atlas for profiling live cells using PETAL. **B**–**F** IHC of human tumor tissues was used to evaluate FK002 expression in various human tumors or corresponding normal organs. Scale bar, 50 μm. The quantification data is shown in right below. Quantitative data are presented as means ± SD of *n* = 30,15,77 or 6 samples. ns not significant; *p < 0.05, ***p < 0.001.

Notably, FK002 demonstrated the highest positivity rate (31%) among all tested antibodies in human lung cancer samples. The FK002 antibody (Antibody number, CL029847) is specifically highly expressed in squamous cell lung carcinoma. The flow chart for constructing a cell surface mAb-target atlas using the PETAL is shown in Fig. 1A.

To evaluate the therapeutic potential of FK002 across various cancer types, we analyzed its expression profile. Using tumor tissue microarrays comprising 23 types of human cancers and corresponding NATs, we assessed FK002 expression by IHC. Our results indicated that FK002 is highly expressed in breast and skin cancers compared to normal tissues (Fig. 1B–F).

### Generation and target validation of FK002 antibody

**Fig. 2:**
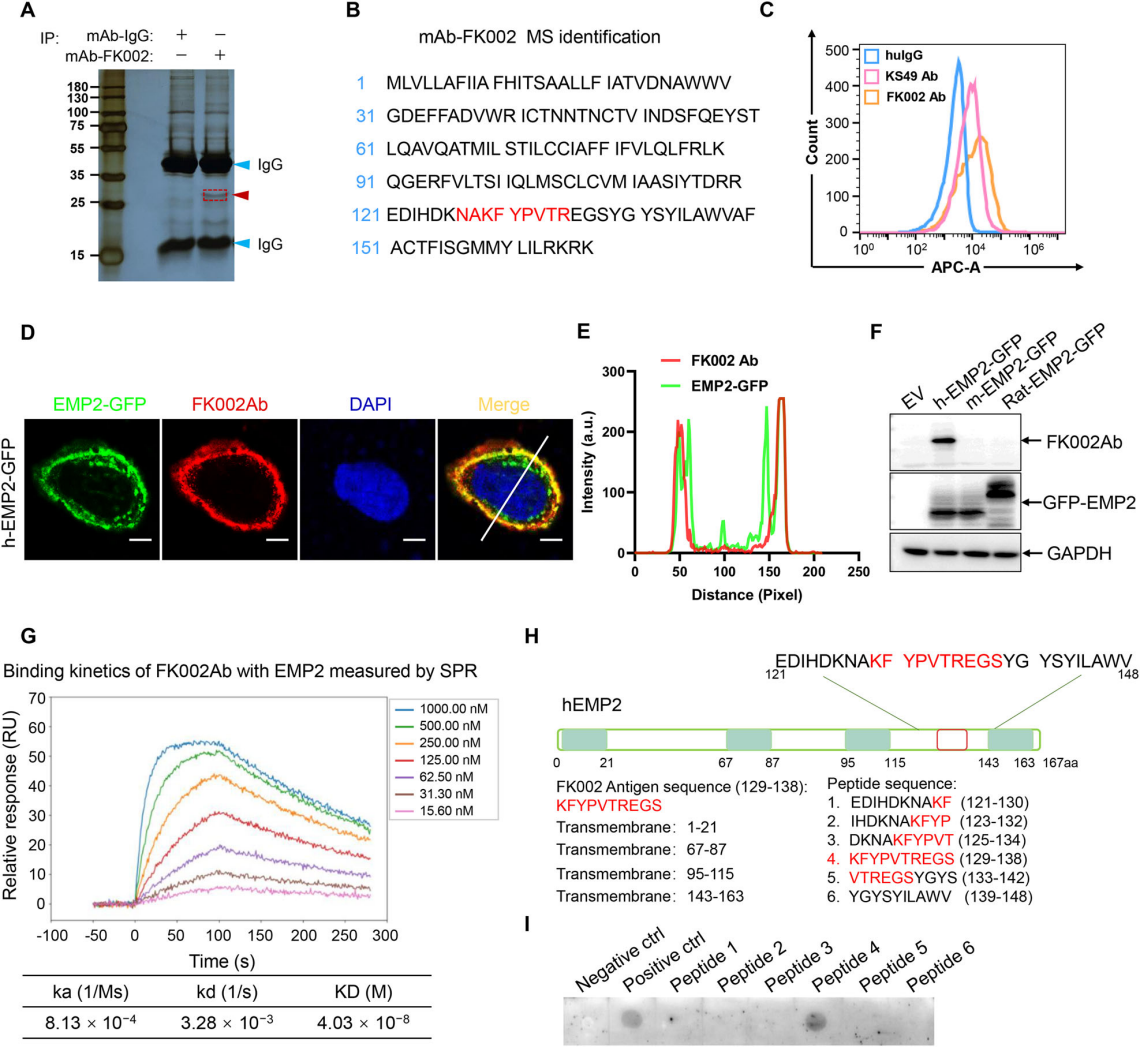
Generation and target validation of FK002 antibody. **A**, **B** Silver staining of anti-FK002 immunoprecipitates is shown in (**A**). Peptides labeled red are ones identified by MS (**B**). **C** Flow cytometry of NCI-H226 cells incubated with IgG, KS49, and FK002 antibody (50 μg/ml) for 30 min. **D**, **E** Immunofluorescence of A549 cells transfected with hEMP2-GFP (**D**). The quantitative analysis of colocalization is shown in (**E**). **F** Immunoblot of anti-FK002 and anti-GFP from HEK293T cells transfected with hEMP2-GFP, mEMP2-GFP, and Rat-EMP2-GFP. **G** A real-time SPR assay showing the association–dissociation curves of surface-immobilized EMP2 protein with FK002 antibody. The data shown are representative of *n* = 3 independent experiments. **H** Six 10-amino acid peptide sequences were designed based on the FK002 antigen sequence. **I** Binding affinity screening of FK002Ab against 8 peptide variants (1 μg/spot). Spot 1: negative control (STING protein); spot 2: positive control (human EMP2 protein); Spots 3~8: FK002-derived peptides.

The antigen sequence of the FK002 antibody is known to be KFYPVTREGS, which can be matched to human EMP2. To verify the target antigen of the FK002 antibody, the FK002 antibody was incubated with NCI-H226 whole-cell lysate for an endogenous co-immunoprecipitation assay. The results of the silver staining experiment showed the target protein bands to which the FK002 antibody specifically binds (Fig. 2A). In addition, we established the target antigen of the FK002 antibody is EMP2 through qualitative mass spectrometry (Fig. 2B). To assess whether the FK002 antibody specifically targets EMP2, we generated a mouse mAb (FK002) and verified it in several assays including flow cytometry, immunofluorescence, and immunoblotting (Fig. 2C–F). Flow cytometry confirmed that the specificity of FK002 is better than the known EMP2 antibody KS49 (Fig. 2C). IF staining and immunoblotting with the FK002 antibody revealed that the FK002 antibody specifically targets human EMP2 (Fig. 2D–F).

In the development of ADCs, the affinity of the antibody is indeed one of the key factors determining the drug’s efficacy and safety. To determine the affinity of the FK002 antibody toward its target antigen EMP2, we performed a real-time surface plasmon resonance (SPR) analysis, measuring the association and dissociation kinetics of FK002 with immobilized EMP2 protein. The data from SPR (kinetic profiling) confirm that the antibody binds to the target antigen with moderate affinity, with an SPR-measured KD value of 4.03 × 10^−^^8^ M (Fig. 2G). To determine the epitope recognized by FK002 antibody, we designed and synthesized six peptides based on the known FK002Ab antigen sequence (KFYPVTREGS) and detected the epitope recognized by FK002 antibody through dot blot (Fig. 2H, I). The data demonstrated that the peptide sequence KFYPVTREGS was identified as the linear epitope for the FK002 antibody.

### Characterization of EMP2-targeted antibody-drug conjugates

**Fig. 3:**
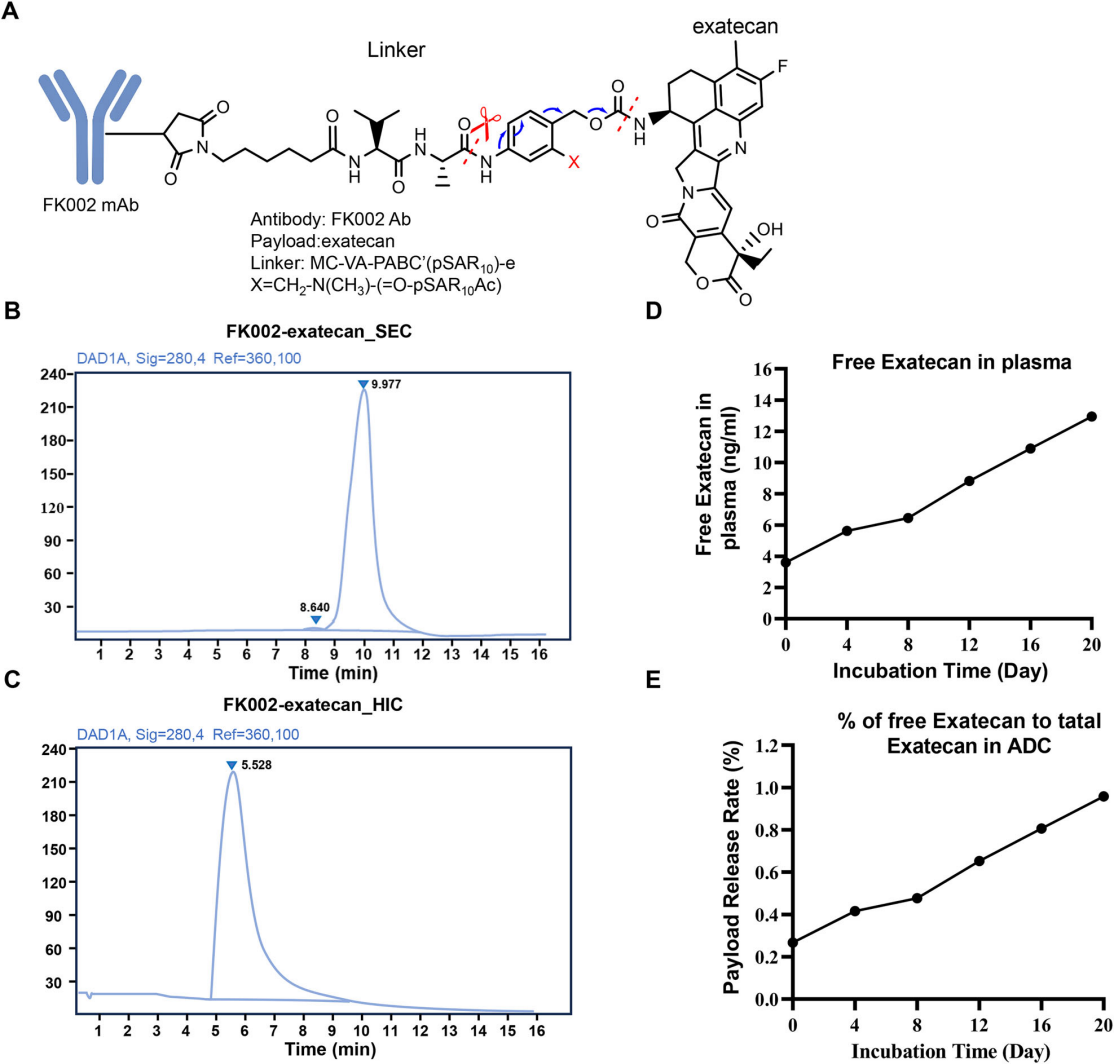
Characterization of EMP2-targeted antibody-drug conjugates. **A** Schematic representation of FK002- exatecan structure. Exatecan was conjugated to FK002 by T1000 (T1000, Mc-VA-PABC(PSAR1o)) linker. **B** SEC-HPLC analysis. **C** HIC-HPLC analysis. The spectrum of FK002-exatecan showed one main peak, representing the coupling of 8 T1000-Exatecan. The DAR of FK002- exatecan was approximately 8. **D** Free Exatecan dissociated from FK002-exatecan in human plasma. FK002- exatecan at 10 μg/mL was incubated with fresh human plasma at 37 °C for 4, 8, 12, 16, and 20 days. The amount of free Exatecan in plasma was detected by LC-MS/MS. **E** Quantitative analysis of percentages of Exatecan dissociated from FK002-exatecan incubated with fresh human plasma at 37 °C for 4, 8, 12, 16, and 20 days.

A schematic representation of the FK002-exatecan structure is shown in Fig. 3A. Exatecan was conjugated to FK002 by T1000 (T1000, Mc-VA-PABC(PSAR1o)) linker. T1000 is a novel linker payload technology used for developing ADCs, which can overcome the difficulty of directly coupling antibodies with exatecan due to its strong hydrophobicity, and endow ADCs with stronger bystander effects and tumor infiltration ability [33]. To clarify the pharmacological characteristics of ADCs, we further assessed the purity and drug loading of ADCs. The SEC-HPLC result displayed that the purity of FK002-exatecan was 98.99% (Fig. 3B). The HIC-HPLC result demonstrated one main peak, representing the coupling of 8 T1000-Exatecan (Fig. 3C). The representative structure of FK002-exatecan with an average of the drug-to-antibody ratio (DAR) at 8:1. FK002-exatecan is stable in human plasma in vitro with less than 2% of exatecan dissociated from the conjugates after incubation up to 20 days (Fig. 3D, E).

### Induction by FK002-exatecan of EMP2 internalization

**Fig. 4:**
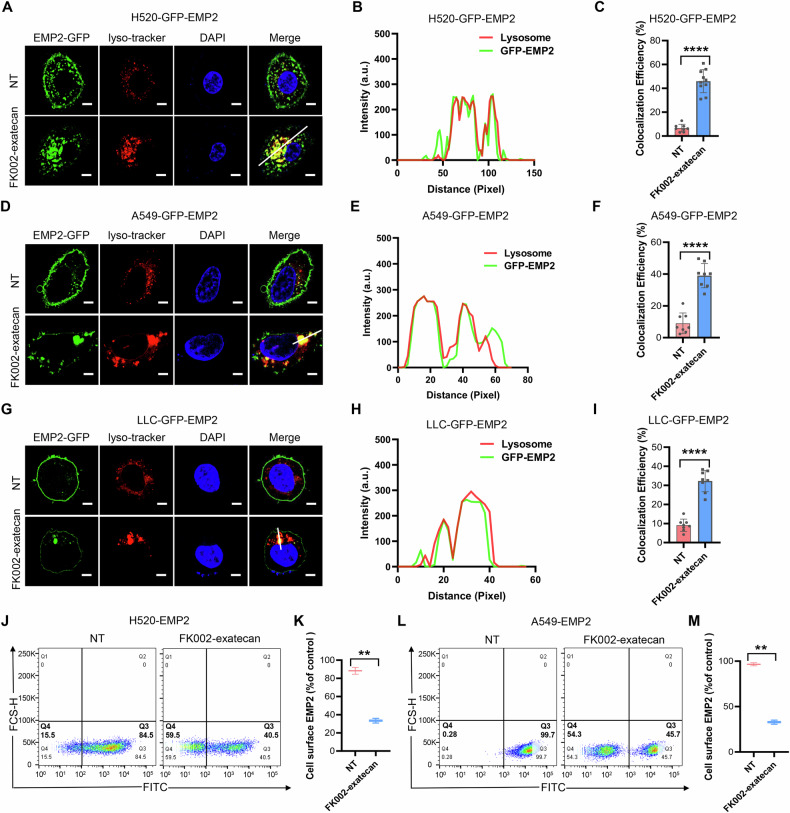
Induction by FK002-exatecan of EMP2 internalization. **A**–**I** Immunofluorescence of EMP2-GFP overexpressing NCI-H520, NCI-A549, and LLC cells exposed to FK002-exatecan (100 nM) for 12 h (**A**, **D**, **G**). Quantitative analysis of colocalization of EMP2 and lysosomes was shown in (**B**, **C**, **E**, **F**, **H**, and **I**). Lysosomes were stained with Lysotracker red (red), and the nucleus was stained with DAPI (blue). **J**–**M** Flow cytometry of EMP2-overexpressing NCI-H520 and NCI-A549 cells incubated with or without FK002-exatecan (100 nM) for 48 h (**J** and **L**). Quantitative analysis of the internalization ability of EMP2 on the cell surface induced by FK002-exatecan was shown in (**K** and **M**). Data are expressed as mean ± SD of *n* = 3 independent experiments (**J**–**M**). **p < 0.01.

The mechanism of action of ADCs is complex, often requiring drug internalization followed by intracellular processing and payload release [34]. To determine FK002-exatecan-induced EMP2 internalization, we constructed hEMP2-GFP fusion overexpressing NCI-A549, NCI-H520, and LLC cell lines (Supplementary Fig. 1H). Immunofluorescence analysis demonstrated EMP2 colocalization with the lysosomal-associated marker (lysotracker) after stimulation with FK002-exatecan, and EMP2 protein on the membrane surface decreased significantly compared to the no-treatment group (Fig. 4A–I). Quantitative analysis also confirmed that FK002-exatecan induced ADC-EMP2 complex internalization (Fig. 4C, F, I). What’s more, flow cytometry was performed to determine the ability of FK002-exatecan to induce EMP2 internalization (Fig. 4J, L). Quantitative analysis confirmed that less than 35% of EMP2 remained on the cell surface after FK002-exatecan stimulation (Fig. 4K, M).

### Induction of cell cycle arrest and apoptosis by FK002-exatecan in vitro

**Fig. 5:**
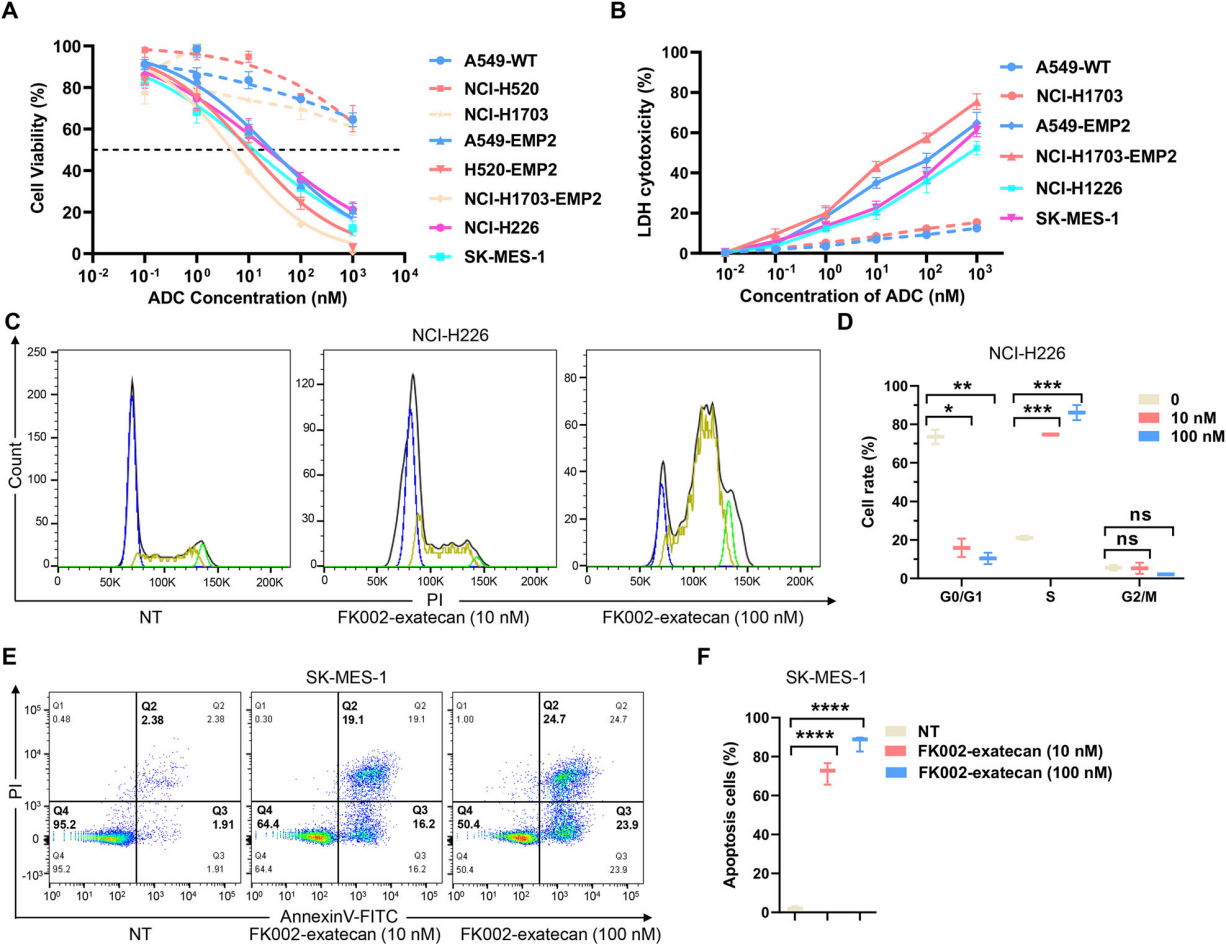
Induction of apoptosis by FK002-conjugated EMP2 protein in vitro. **A** Cell viability assay of lung cancer cells with high EMP2 expression exposed to FK002-exatecan (0, 0.1, 1, 10, 100, 1000 nM) for 48 h. **B** Cytotoxicity assay of lung cancer cells with high EMP2 expression exposed to FK002-exatecan (0, 0.1, 1, 10, 100, 1000 nM) for 48 h. **C** and **D** Cell cycle kinetic analysis of NCI-H226 cells treated with FK002-exatecan (0, 10, 100 nM) for 12 h. Quantitative data are shown in (**D**). **E** and **F** Flow cytometry of SK-MES-1 cells treated with FK002-exatecan (0, 10, 100 nM) for 48 h. Quantitative data are shown in (**F**). Data are expressed as mean ± SD of *n* = 3 independent experiments (**A**–**F**). ns not significant; *p < 0.05, **p < 0.01, ***p < 0.001, ****p < 0.0001.

EMP2 has been reported as an oncogene in invasive breast cancer and endometrial carcinoma, and is expected to be a novel target for cancer treatment [14, 29]. To evaluate the effect of EMP2 on lung cancer cells, we performed cell proliferation, clonal formation, and migration assays in wild-type and EMP2-overexpressing lung cancer cells. The results showed that EMP2 had limited effects on the proliferation, clonal formation, and migration of lung cancer cells (Supplementary Fig. S1B–G). To evaluate the cytotoxicity of FK002-exatecan in vitro, we screened lung cancer cell lines with high EMP2 expression by immunoblotting. The result suggested that PC9, NCI-H226, and SK-MES-1 showed high expression of EMP2 (Supplementary Fig. 1A). We employed wild-type and EMP2-overexpressing lung cancer cells, including A549, H520, NCI-H1703, and lung cancer cell lines with high EMP2 expression, NCI-H226 and SK-MES-1, to test the cytotoxicity of FK002-exatecan. We found FK002-exatecan specifically kills tumor cells with high EMP2 expression in a dose-dependent manner (Fig. 5A, B). We further explored whether FK002-exatecan triggers apoptosis by using an Annexin V/PI assay. Tumor cells were incubated with 10 nM and 100 nM of FK002-exatecan for 24 h. Cells treated with PBS were used as a negative control. FK002-exatecan induced apoptosis in lung cancer cells with high EMP2 expression compared to the negative control (Fig. 5E, F, and Supplementary Fig. 2C–F). Mechanically, Flow cytometry analysis demonstrated that FK002-exatecan induced cell cycle arrest of tumor cells in the DNA synthesis (S) phase (Fig. 5C, D, and Supplementary Fig. 2A, B). Overall, these results indicated that FK002-exatecan significantly induced cell cycle arrest and increased apoptosis in EMP2-overexpressing tumor cells by targeting EMP2.

### Antitumor activity of FK002-exatecan of EMP2 by targeting EMP2

**Fig. 6:**
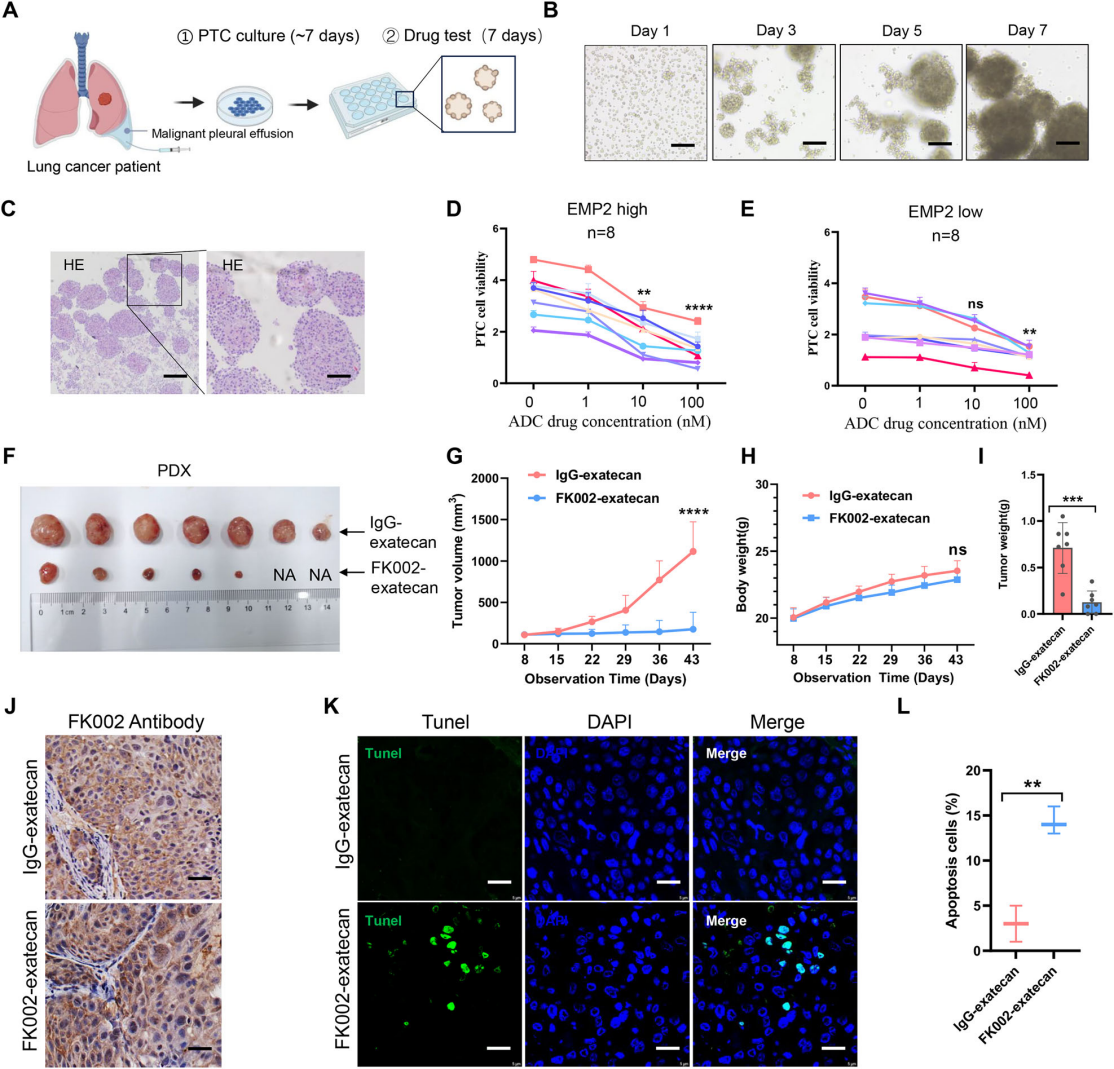
Antitumor activity of FK002-exatecan on patient-derived microtumors and patient-derived xenografts. **A** Timeline of PTC's culture generation and drug efficacy testing. **B** Bright-field images depicting representative phenotypes of PTCs from one lung cancer patient at the indicated times. Scale bars, 100 μm. **C** Hematoxylin and eosin staining (HE) of the derivative PTCs. Scale bar, 100 μm. The drug-response profile of PTCs from 8 lung cancer patients with high EMP2 expression (**D**) and 8 with low EMP2 expression (**E**). ns not significant; **p < 0.01; ****p < 0.0001. **F**–**I** Mice with NSCLC patient-derived xenograft tumors were treated with a single dose of 10 mg/kg FK002-exatecan or IgG-exatecan once per week for the indicated times. Tumor size (**F**), Tumor growth curve (**G**), Body weight (**H**), and Tumor weight (**I**) of tumors from PDX models were displayed. Quantitative data are presented as means ± SD of seven mice. Independent Student’s *t*-test. ns not significant; ***P < 0.001; ****p < 0.0001. **J** IHC staining of tumor tissues from PDX models. Scale bars, 100 μm. **K**, **L** Tunel staining of tumor tissues from PDX models. Scale bars, 100 μm; Quantitative data are presented as means ± SD of four mice. Independent Student’s *t*-test. **P < 0.01.

Next, we evaluated the antitumor activity of FK002-exatecan of EMP2 in a series of lung cancer models in vivo. Patient-derived tumor-like cell clusters (PTCs) result from the self-assembly and proliferation of primary epithelial, fibroblast, and immune cells, which structurally and functionally recapitulate original tumors [35]. We previously employed PTC to assess drug efficacy for lung cancer, and this model has demonstrated high accuracy in predicting the effects of chemotherapy [36]. PTCs achieve testing drug efficacy within 2 weeks after obtaining the malignant pleural effusion (Fig. 6A, B). In total, PTCs from 16 patients with lung cancers were generated to evaluate the antitumor effect of FK002-exatecan. HE staining demonstrated that the PTCs exhibited high morphological similarities with the primary lung cancer (Fig. 6C). The expression of EMP2 in the PTCs was also detected by IHC staining. Of them, 8 PTCs had high EMP2 expression, and 8 PTCs had low EMP2 expression. The statistical significance of drug efficacy among different treatment arms was determined by a paired *t*-test. In the group of high EMP2 expression, we observed that FK002-exatecan effectively restrains tumor growth in PTCs compared to the control group, and this effect was concentration-dependent. but not when EMP2 expression was low or undetectable (Fig. 6D, E). Secondly, we established patient-derived tumor xenograft (PDX) models from lung cancer. Treatment with FK002-exatecan remarkably reduced tumor growth in mice compared to the IgG-exatecan control group (Fig. 6F–I and Supplementary Fig. 3A). During this period, the mice displayed no significant changes in body weight, indicating the ADC drugs had no significant side effects (Fig. 6H). The expression of EMP2 in the PDXs was detected by IHC staining (Fig. 6J). In addition, by tunel staining, we found that FK002-exatecan effectively induces apoptosis of lung cancer cells with high EMP2 expression (Fig. 6K, L).

**Fig. 7:**
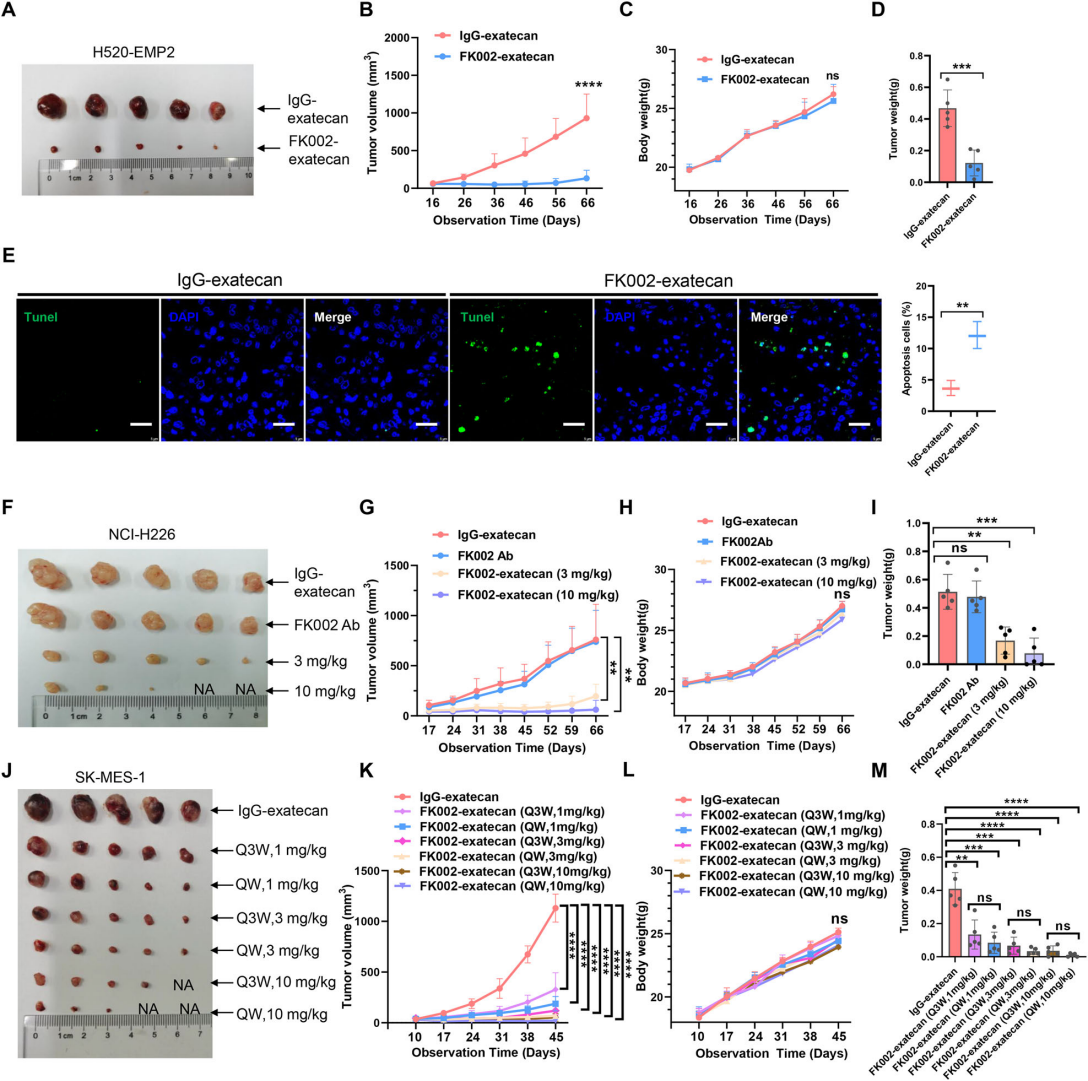
Antitumor activity of FK002-exatecan on xenograft tumors. **A**–**D** Mice with EMP2-overexpressing NCI-H520 xenograft tumors were treated with a single dose of 10 mg/kg FK002-exatecan or IgG-exatecan once per week for the indicated times. Tumor size (**A**), Tumor growth curve (**B**), Body weight (**C**), and Tumor weight (**D**) of tumors from xenograft models were displayed. Quantitative data are presented as means ± SD of five mice. Independent Student’s *t*-test. ns not significant; ***P < 0.001; ****p < 0.0001. Tunel staining and quantitative data of tumor tissues from xenograft models are shown in (**E**). Scale bars, 100 μm. Independent Student’s *t*-test. **P < 0.01. **F**–**I** Mice with NCI-H226 xenograft tumors were treated with FK002Ab, low-dose FK002-exatecan (3 mg/kg), high-dose FK002-exatecan (10 mg/kg), or IgG-exatecan once per week for the indicated times. Tumor size (**F**), Tumor growth curve (**G**), body weight (**H**), and Tumor weight (**I**) of tumors from xenograft models were displayed. Quantitative data are presented as means ± SD of five mice. Independent Student’s *t*-test. ns not significant; **p < 0.01; ***P < 0.001. **J**–**M** Mice with SK-MES-1 xenograft tumors were treated with low-frequency FK002-exatecan (Q3W, 1 mg/kg, 3 mg/kg, 10 mg/kg), high-frequency FK002-exatecan (QW, 1 mg/kg, 3 mg/kg, 10 mg/kg), or IgG-exatecan for the indicated times. Tumor size (**J**), Tumor growth curve (**K**), body weight (**L**), and Tumor weight (**M**) of tumors from xenograft models were displayed. Quantitative data are presented as means ± SD of five mice. Independent Student’s *t*-test. ns not significant; **p < 0.01; ***P < 0.001; ****p < 0.0001.

Furthermore, we found a similar suppressive effect of FK002-exatecan on the growth of xenografted H520-EMP2, NCI-H226, SK-MES-1, and PC9 cells, which, along with reduced tumor weight compared to the IgG-exatecan control group (Fig. 7A–M and Supplementary Fig. 4A–E). To more accurately evaluate the antitumor efficacy of the ADC, we employed SK-MES-1 xenograft models treated with a lower dose (1 mg/kg) and low-frequency (administered every three weeks) dosing regimens. The results demonstrated that reduced doses and dosing frequency administration still exhibited significant antitumor efficacy (Fig. 7J–M and Supplementary Fig. 4I). The results suggested that even at reduced dosing frequency, the ADC retained significant tumor-suppressive activity, further confirming its target specificity and sustained pharmacological effect. Collectively, our findings substantiate the therapeutic feasibility of reduced-frequency ADC administration for effective tumor suppression. The tumor growth curve of tumors from xenograft models was displayed in Supplementary Fig. 4F–I. Moreover, tunel staining confirmed that FK002-exatecan effectively induces apoptosis of tumor cells with high EMP2 expression in the ADC administration group (Fig. 7E and Supplementary Fig. 4E), indicating that FK002-exatecan could inhibit tumor growth by inducing apoptosis of tumor cells. Taken together, these findings indicate that FK002-exatecan of EMP2 has effective antitumor activity against lung cancer, especially lung squamous cell carcinoma.

## Discussion

ADCs have emerged as a credentialled class of anticancer drugs for both solid and hematological malignancies, with regulatory approvals mainly as single agents. Despite extensive preclinical and clinical efforts to develop rational ADC-based combinations, to date, only a few have shown significant survival benefits compared to standard of care. Hence, the exploration of novel ADC targets is one of the most compelling areas of research in the field. In our study, by using a tissue-microarray-based screening of an antibody library, we developed the EMP2-directed ADC, FK002-exatecan, which induced tumor eradication in several lung cancer cell lines and xenograft mouse models. We provide one of the first examples of ADCs targeting EMP2, offering a novel strategy for lung cancer therapy.

In contrast to lung adenocarcinoma, patients afflicted with lung squamous cell carcinoma (LSCC) have not benefited from targeted therapies [37]. Although immunotherapy has significantly improved cancer patients’ outcomes, the relatively low response rate and severe adverse events hinder the clinical application of this promising treatment in LSCC [38, 39]. ADC preferentially targets tumor cells expressing specific targets and offers a novel therapeutic tool for the management of patients with LSCC. Our data suggest that EMP2 is more frequently expressed in squamous lung cancer than other pathologic types of lung cancer. Moreover, we also found that EMP2 is more likely to be expressed in squamous carcinoma in other organs, such as squamous skin carcinoma, squamous cervical carcinoma, etc. Our in vivo data showed that EMP2-directed ADC was highly effective in the treatment of LSCC, and further preclinical studies or early-phase clinical trials are worthwhile. Similarly, other molecules, which are overexpressed in NSCLC cells, can be targeted by some ADCs that are currently under investigation. For instance, targeting HER2 [e.g., trastuzumab emtansine (T-DM1), NCT02289833], trophoblast cell surface antigen 2 (Trop-2) [e.g., sacituzumab govitecan (IMMU-132), NCT01631552], mesenchymal-epithelial transition (c-MET) [e.g., telisotuzumab vedotin (ABBV-399), NCT03574753], protein tyrosine kinase 7 (PTK7) (e.g., PF-06647020, NCT02222922). While ADCs have yet to receive approval for lung cancer treatment, preclinical data and phase I/II clinical trials show promising outcomes in this context.

EMP2 has emerged as a novel prognostic and predictive biomarker in various human cancers. Previous efforts in developing EMP2-targeting therapies, such as the granzyme B fusion protein GrB-Fc-KS49, have demonstrated promising therapeutic potential for targeted breast cancer treatment [40]. ONCR-201, a fully human monoclonal antibody against EMP2 developed by OncoResponse, Inc., is currently in preclinical development as an anticancer therapy. These prior studies demonstrated the therapeutic potential of EMP2-targeting strategies in breast, ovarian, and endometrial cancers [15, 40, 41].

Building upon the anti-EMP2 antibody, we have developed EMP2-directed ADC conjugated with the topoisomerase I inhibitor exatecan. This novel ADC (FK002-exatecan) demonstrates superior targeting specificity, enhanced cellular internalization, and improved therapeutic efficacy compared to unconjugated anti-EMP2 antibodies.

Upon intravenous administration, EMP2-directed ADCs circulate, reach tumor sites, and bind to tumor-associated antigens. Subsequently, the antigens undergo endocytosis, internalizing the ADCs into tumor cells, where they are delivered to lysosomes, releasing exatecan. This released exatecan exerts cytotoxic effects by inducing apoptosis through DNA damage, eliminating nearby cancer cells via the bystander effect. However, challenges arise in circulation due to potential toxicity or side effects from premature exatecan release or inadvertent ADC binding to normal tissues. Non-target tissues and organs, particularly those with robust blood flow and phagocytic activity, may capture ADCs [42]. Moreover, the dispersion of ADCs in normal cells expressing the target antigen can lead to side effects or treatment ineffectiveness. Despite the overexpression of EMP2 in tumors, its detection in normal tissues raises safety concerns. In our study, we noted no weight loss or apparent toxicity symptoms during EMP2-directed ADC treatment. Nonetheless, the studies in primates (such as the crab-eating macaque) are especially important to establish the in vivo safety profile because the lack of cross-reactivity of the antibody does not allow an on-target tox assessment in mouse models.

To maximize the therapeutic potential of FK002-exatecan while minimizing its off-target effects, further optimization of the FK002 antibody’s affinity is required. Computational modeling (AlphaFold) is employed to predict complementarity-determining region CDR mutations for sub-nanometer (sub-nM) affinity while avoiding on-target off-tumor binding. Subsequently, combine deep mutational scanning with high-throughput SPR screening to identify a high-affinity CDR mutation of the FK002 antibody.

Several ADCs have been investigated in clinical trials, and additional research efforts are ongoing, aimed at identifying novel target antigens and evaluating both rationally-designed ADCs and new combinations with other anticancer drugs. In particular, some clinical trials are currently investigating ADCs in combination with immune checkpoint inhibitors (NCT03288545 for urothelial cancer; NCT05115500 for solid tumor; NCT04681131 and NCT05609968for lung cancer; NCT06103864 for breast cancer), based on preclinical evidence suggesting that ADCs can boost immune cell infiltration in tumors, triggering robust antitumor responses [43]. The mechanisms involved are diverse, ranging from inducing immunogenic cell death, promoting dendritic cell maturation, and increasing T lymphocyte infiltration, to reinforcing immunological memory and influencing the expression of immune-regulatory proteins like programmed death ligand 1 (PD-L1) and major histocompatibility complex (MHC) [44–46]. Several ADCs demonstrated greater efficacy in preclinical models with intact immune systems, supporting the relevance of their immunomodulatory functions [45, 46]. These findings may present a rationale for designing clinical trials using low doses of ADCs as immunostimulants to enhance the activity of immunotherapy.

## Method details

### Animals

6–8-week-old male BALB/c nude mice were purchased from Shanghai Laboratory Animal Center, CAS, China. and housed in specific pathogen-free (SPF) facilities at the Laboratory Animal Center of Tongji University. Sex- and age-matched animals aged 6–8 weeks were used in all experiments unless otherwise specified. BALB/c nude mice were randomly divided into the following two or three treatment groups: PBS control (PBS) group; low-dose ADC (from Abmart) treatment group (ADC: 3 mg/kg); high-dose ADC treatment group (ADC: 10 mg/kg). Sample sizes were determined based on power analysis (α = 0.05, power = 0.8) using effect sizes from pilot experiments (*n* = 5/group). Animals with abnormal baseline behavior or weight loss >20% were excluded. Criteria were pre-defined in the study protocol. Investigators were blinded to treatment groups during data collection and analysis. All animal experiments were approved by the Tongji University School of Medicine Animal Care and Use Committee and were conducted following the National Institutes of Health Guidelines for the Care and Use of Laboratory Animals.

### Clinical samples

Human normal or cancer tissue samples were obtained from the Shanghai Pulmonary Hospital, with approval from the Shanghai Pulmonary Hospital Institutional Review Board. Tissue samples were used for FK002 antibody immunohistochemical staining. Inclusion criteria for clinical samples: Patients with NSCLC (stage I-IIIA) whose tumors had been surgically removed were enrolled, and patients receiving neoadjuvant therapy before surgery were excluded. We collected a total of 80 clinical samples that met the standards. Informed consent was obtained from all subjects.

### Cell culture

Human embryonic kidney epithelial cells (HEK293T; ATCC CRL-11268), mouse Lewis lung carcinoma cells (LLC; ATCC CRL-1642), and lung adenocarcinoma cells (PC9; ECACC 90071810) were cultured in Dulbecco’s Modified Eagle’s Medium (DMEM; Gibco) supplemented with 10% (v/v) heat-inactivated fetal bovine serum (FBS; Gibco), 1% (v/v) penicillin-streptomycin, 1 mM of sodium pyruvate, 2 mM of L-glutamine, 10 mM of HEPES buffer, and 50 μM of 2-mercaptoethanol (all from Gibco). Human non-small cell lung cancer cells (A549; ATCC CCL-185), Human lung squamous cell carcinoma cells (NCI-H520; ATCC HTB-182), human lung squamous cell carcinoma cells (NCI-H226; ATCC CRL-5826) and human lung squamous cell carcinoma cells (NCI-H1703; ATCC CRL-5889) were cultured in Roswell Park Memorial Institute (RPMI; Gibco) supplemented with 10% (v/v) heat-inactivated fetal bovine serum (FBS; Gibco), 1% (v/v) penicillin-streptomycin, 1 mM of sodium pyruvate, 2 mM of L-glutamine, 10 mM of HEPES buffer, and 50 μM of 2-mercaptoethanol (all from Gibco). Human lung squamous cell carcinoma cells (SK-MES-1; ATCC HTB-58) were cultured in minimum essential medium (MEM; Procell) supplemented with 10% (v/v) heat-inactivated fetal bovine serum (FBS; Gibco), 1% (v/v) penicillin-streptomycin, 1 mM of sodium pyruvate, 2 mM of L-glutamine, 10 mM of HEPES buffer, and 50 μM of 2-mercaptoethanol (all from Gibco). All cell lines used in this study were obtained from ATCC or ECACC. Each cell line was authenticated within the past 12 months using short tandem repeat (STR) profiling to confirm species origin and absence of cross-contamination. Additionally, all cultures were routinely tested for mycoplasma contamination via commercial kits (D101-02, Vazyme), and only confirmed negative samples were used in experiments.

### Generation process of FK002 mAb

First, the peptide antigen of FK002 was designed to be a 10-amino-acid sequence. Then, the peptide antigens (KFYPVTREGS) were chemically synthesized (GL Biochem, Shanghai), with purity and molecular weight verified by high-performance LC and MS. To construct the FK002 mAb, the peptide antigen (40 μg) was emulsified 1:1 with complete Freund’s adjuvant (F5881-10ML, Sigma) and administered to 6–8-week-old mice via intraperitoneal injection. This was followed by four booster immunizations at two-week intervals using the same formulation. Seven to ten days after the final immunization, serum was collected for antibody titer measurement by ELISA. The mouse showing the highest antibody titer (≥1:10,000) was selected for splenocyte isolation. Three to four days after the last boost, splenocytes were harvested and fused with myeloma cells using PEG-mediated cell fusion to generate hybridomas. Each hybridoma cell line was used to prepare ascites containing 1–20 mg of mouse immunoglobulin Gs (IgGs) with varying concentrations from 0.1 to 10 mg/ml. FK002 mAb was then sequenced to obtain the V region and designed into a chimeric antibody format by using human IgG1κ, subsequently generated in Chinese Hamster Ovary cells (CHO) expression system and purified by Mabselect Sure Resin (GE HealthCare) in a gravity column. Detailed steps for the construction of FK002 mAb (from Abmart) were described in a previous study [32].

The sequence of the FK002 antibody is displayed as follows:

Heavy chain (HC):

DVQLQESGPGLVKPSQSLSLTCSVTGYSITSGYYWNWIRQFPGNKLEWMGYIRYDGRNNYNPSLKNRISITRDTSKNQFFLKLNSVTTEDTATYYCARDFDYWGQGTTLTVSSASTKGPSVFPLAPSSKSTSGGTAALGCLVKDYFPEPVTVSWNSGALTSGVHTFPAVLQSSGLYSLSSVVTVPSSSLGTQTYICNVNHKPSNTKVDKKVEPKSCDKTHTCPPCPAPELLGGPSVFLFPPKPKDTLMISRTPEVTCVVVDVSHEDPEVKFNWYVDGVEVHNAKTKPREEQYNSTYRVVSVLTVLHQDWLNGKEYKCKVSNKALPAPIEKTISKAKGQPREPQVYTLPPSRDELTKNQVSLTCLVKGFYPSDIAVEWESNGQPENNYKTTPPVLDSDGSFFLYSKLTVDKSRWQQGNVFSCSVMHEALHNHYTQKSLSLSPGK

Light chain (LC):

DIVLTQSPASLAVSLGQRATISCRASESVDTYGNSFMHWYQQKPGQPPKLLIYRASNLESGIPARFSGSGSRTDFTLTINPVEADDVATYYCHQSNEDPDTFGGGTKLEIKRTVAAPSVFIFPPSDEQLKSGTASVVCLLNNFYPREAKVQWKVDNALQSGNSQESVTEQDSKDSTYSLSSTLTLSKADYEKHKVYACEVTHQGLSSPVTKSFNRGEC

### ADC preparation

A solution of naked mAb (10 mg/mL in PBS (PH = 7.0) + 2 mmol/L EDTA) was treated with 7 molar equivalents of tris(2-carboxyethyl) phosphine (TCEP) for 2 h at 37 °C. Then 14 molar equivalents of the linker drug were added to the fully reduced antibody while keeping residual T1000 (pSAR) concentration below 10% (v/v). The mixture was incubated at room temperature for an hour, followed by purification over a Zeba spin desalting column (Thermo Fisher Scientific) to remove excess reagents. In this step, the resulting ADC was also buffer exchanged into formulation buffer (20 mmol/L histidine, 150 mmol/L NaCl, pH 5.5). Moreover, the excess linker drug could be removed thoroughly by treating the product with activated charcoal (30 mg of charcoal to 1 mL ADC solution), followed by vortexing for 2 h at room temperature. The charcoal was then removed via sterile filtration (0.2 μm PES filters), and the final ADC was stored at −80 °C.

### ADC concentration and DAR measurement

The concentration of mAb and the conjugated drug in the ADC was calculated by measuring UV absorbance of an aqueous solution of the ADC at two wavelengths of 280 nm and 370 nm, as the total absorbance at any given wavelength is equal to the sum of the absorbance of all light-absorbing chemical species that are present in a system (additivity of absorbance). The equations were as follows:

1 $${{\rm{A}}}_{280}={{\rm{A}}}_{{\rm{D}},280}+{{\rm{A}}}_{{\rm{A}},280}={{\rm{\varepsilon }}}_{{\rm{D}},280}{{\rm{C}}}_{{\rm{D}}}+{{\rm{\varepsilon }}}_{{\rm{A}},280}{{\rm{C}}}_{{\rm{A}}}$$

2 $${{\rm{A}}}_{370}={{\rm{A}}}_{{\rm{D}},370}+{{\rm{A}}}_{{\rm{A}},370}={{\rm{\varepsilon }}}_{{\rm{D}},370}{{\rm{C}}}_{{\rm{D}}}+{{\rm{\varepsilon }}}_{{\rm{A}},370}{{\rm{C}}}_{{\rm{A}}}$$

The values of ε_A,280_, ε_A,370_, ε_D,280_, and ε_D,370_ were estimated based on the calculation of the amino acid sequence of the antibody or obtained by UV measurement of the compound according to the Lambert–Beer law. Simultaneous equations (1) and (2) can be solved by substitution of the above values, and then C_A_ and C_D_ were determined. Dividing C_D_ by C_A_, the average number of conjugated drug molecules per antibody was determined.

ADCs with different numbers of drugs per antibody were separated using a butyl HIC column (TSK-gel Butyl-NPR 4.6 × 35 mm 2.5 μm, Tosoh Bioscience) at room temperature. HIC was also performed on an Agilent 1260 Infinity II HPLC system with UV detection at 280 nm. Mobile phase A was 1.5 mol/L (NH_4_)_2_SO_4_, 50 mmol/L K_2_HPO_4_, pH 7.0, and mobile phase B was 21.3 mmol/L KH_2_PO_4_, 28.6 mmol/L K_2_HPO_4_, 25% (v/v) isopropanol, pH 7.0. The gradient program was as follows: B%:

0–25% (0–1 min, 0.8 mL/min), 25% (1–3 min, 0.6 mL/min), 25–80% (3–13 min, 0.6 mL/min), 80% (13–17 min, 0.6 mL/min), 80–0% (17–17.10 min, 0.5 mL/min), and 0% (17.10–25 min, 0.7 mL/min).

### Flow cytometry

For analysis of the specificity of the FK002 antibody, NCI-H226 cells were trypsinized, rinsed in cold PBS, and labeled with human anti-IgG mAb, human anti-EMP2 mAb (KS49), or mouse anti-EMP2 mAb (FK002Ab) in PBS at 4 °C for 30 min. Then, cells were rinsed with PBS for 2~3 times and incubated with FITC-conjugated goat anti-human IgG (WE0362-GMS, Biolab) or Alexa Fluor® 488 Conjugated goat anti-mouse IgG secondary antibodies (#89853, Cell Signaling Technology), rinsed again, and single-cell suspensions were made using 70 mm cell strainers (BD Biosciences). Finally, cell samples were analyzed on a FACS Calibur flow cytometer (Becton Dickinson). For analysis of the cell cycle status, cells were collected, rinsed in cold PBS, and immobilized with ice-cold 70% ethanol overnight. Cells were rinsed with PBS and incubated with the propidium staining solution for 30 min at room temperature. The propidium staining solution was configured according to the cell cycle analysis kit (C1052, Beyotime Biotechnology). Rinsed again and analyzed on a FACS Calibur flow cytometer (Becton Dickinson).

### Immunoprecipitation and Western blot

Cells were lysed using RIPA Lysis Buffer (Beyotime Biotechnology, China) containing protease inhibitor cocktail (P8340, Sigma–Aldrich) and phosphatase inhibitor cocktail (P5726, Sigma–Aldrich) for 30 min. The cell lysates were centrifuged at 12,000 rpm for 10 min, and the supernatant was collected. For immunoprecipitation, cell lysates were incubated with Anti-FLAG M2 Affinity Gel (A2220, Sigma–Aldrich) at 4 °C overnight. For immunoblotting, the cell lysates or precipitates were denatured in 1× sodium dodecyl sulfate (SDS) protein sample buffer at 95 °C for 10 min and then were resolved by electrophoresis through an 8% or 10% SDS-polyacrylamide gel. Separated proteins were transferred onto polyvinylidene difluoride membranes (PVDF) (ISEQ00010, Sigma–Aldrich). Then, the PVDF membranes were incubated with the prespecified antibodies at the indicated dilutions, and an enhanced chemiluminescence reagent (Thermo Fisher Scientific) was applied for immunoblotting.

### SPR

SPR measurements were performed on a Biacore 8 K instrument (GE HealthCare, Piscataway, NJ, USA). Purified EMP2 protein (18 µg/ml, pH = 8.1) (approximately 12,000 RU) was immobilized on an s-series sensor chip (GE HealthCare, Piscataway, NJ, USA) following standard amine coupling procedures. PBS (BR100672, pH 7.2–7.4, Cytiva) containing 1% dimethyl sulfoxide was used as the running buffer for immobilization. After immobilization, dilute FK002Ab with the same analyte buffer to 8 concentrations (0.02–1) μM. FK002Ab is injected into channel Fc1–Fc2 at a flow rate of 20 μL/min for an association phase of 100 s, followed by 180 s dissociation. The association and dissociation processes are all handled in the analyte buffer. Repeat 8 cycles of analyte according to the analyte concentrations in ascending order. After each cycle of interaction analysis, the chip needs to be regenerated. The final graph was obtained by subtracting the grams of the blank sensor. Experimental data were collected and analyzed using Biacore 8K Manager software (GE HealthCare, Piscataway, NJ, USA) to fit a suitable binding model to obtain an equilibrium dissociation constant of 4.03 × 10^–^^8^ M (Kd).

### Dot blot

The negative control sample (Novoprotein), positive control sample (Cusabio), and the six synthetic peptide samples (GenScript) were diluted in PBS to a final concentration of 1 μg/mL. Next, pipetted 2.5 µL of each sample directly onto the nitrocellulose membrane (#HATF00010, Millipore) in small dots and allowed spots to air-dry 20 min. Incubated the membrane in blocking solution (5% milk in TBST) for 1 h at RT and then incubated the membrane with FK002 antibody for 2 h overnight at 4 °C. After washing with TBST three times, the incubated membrane was incubated with HRP-conjugated mouse secondary antibody (#0712027, Cell Signaling Technology) for 1 h at RT with shaking, followed by washing with TBST three times. Finally, an enhanced chemiluminescence reagent (Thermo Fisher Scientific) was applied for immunoblotting.

Negative control sample: Sumo-STING(139-379aa) protein

Positive control sample: EMP2(116-142aa) protein

Six synthetic peptide samples (From human EMP2):

Peptide 1. EDIHDKNAKF (121-130aa)

Peptide 2. IHDKNAKFYP (123-132aa)

Peptide 3. DKNAKFYPVT (125-134aa)

Peptide 4. KFYPVTREGS (129-138aa)

Peptide 5. VTREGSYGYS (133-142aa)

Peptide 6. YGYSYILAWV (139-148aa)

### Immunohistochemistry (IHC)

EMP2 expression was assessed in formalin-fixed, paraffin-embedded matched pairs of lung cancer tumor tissue and adjacent normal tissue specimens. Two serial 4-mm-thick formalin-fixed (FFPE) paraffin-embedded tissue sections were prepared from each tumor and adjacent normal tissue specimens. Tissue sections were incubated with FK002Ab primary antibody overnight at 4 °C and horseradish peroxidase-conjugated secondary antibody for 1 h at room temperature, followed by immersion in DAB-peroxidase substrate solution and counterstained with hematoxylin.

### A549-EMP2-GFP, H520-EMP2-GFP and LLC-EMP2-GFP reconstituted cells

pCDH-EMP2-GFP lentivirus particles were obtained by co-transfecting pCDH-EMP2-GFP with packaging plasmids (pMD2.G and psPAX2) into HEK293T cells. Following 48 h post-transfection, the culture medium containing pCDH-EMP2-GFP lentivirus particles was collected. A549 cells were infected with concentrated pCDH-EMP2-GFP lentivirus particles. Following 24 h post-infection, cells were then screened with puromycin (1 μg/mL) to obtain stable human EMP2-GFP-overexpressing A549 cells, and a single-cell clone was acquired through serial dilutions in a 96-well plate. The stable cells were confirmed by Western blot and used for further experiments.

hEMP2-overexpressing A549, LLC, NCI-H520, and NCI-H1703 cell lines were constructed in the same methods as described above.

### Apoptosis TUNEL staining

For analysis of the apoptosis of tumor tissue from mice, tissue sections were obtained according to a standard procedure. Then, tissue sections were incubated with 100 µl TUNEL reaction mixture (or 100 µl control label solution for negative control) at 37 °C for 1 h, rinsed in PBS 3 times, and labeled with anti-FITC-AP conj. (“converter-AP”) at 37 °C for 30 min, rinsed again, and incubated with substrate solution in darkness for 10 min at room temperature, and stopped color reaction by adding the ddH2O water. Tunel staining samples were observed with laser scanning confocal microscopy (Leica Microsystems, Buffalo Grove, IL). Detailed steps for tunel staining were described in a previous study.

### Immunofluorescence assay

Cells were seeded in 15 mm glass-bottom cell culture dishes (NEST, Jiangsu, China), and then the cells were treated with ADCs at the indicated dilutions for 12 h. Cells were fixed with 4% paraformaldehyde (PFA) for 30 min at room temperature and permeabilized with 0.1% Triton X-100 for 10 min, followed by blocking with 3% BSA(PBS containing 1% bovine serum albumin (BSA)) at 37 °C for 1 h. Cells were then incubated with primary antibody overnight at 4 °C and labeled with secondary antibody for 1 h at room temperature. Nuclei were counterstained with Hoechst 33342 or DAPI. Images were collected using a Leica TCS SP8 confocal laser microscopy system (Leica Microsystems, Buffalo Grove, IL).

### Migration and proliferation assays

Transwell chambers were used to evaluate cell migration ability. Cells (3 × 10^4^ cells/well) were suspended in a serum-free medium and then seeded into the upper chamber with an 8.0 µm pore (Corning, USA), the lower chamber contained 10% fetal bovine serum (FBS). Subsequently, cells were incubated at 37 °C for 48 h, and cells that migrated through the pores were fixed with 4% paraformaldehyde and stained with 0.1% crystal violet. Cell proliferation was measured using the Cell Counting Kit-8 assay (Dojindo Molecular Tech Inc., Japan) following the manufacturer’s instructions.

### Cytotoxicity assays

Cells were seeded in 96-well plates (NEST, Jiangsu, China) and pretreated with ADCs at a gradient dilution, and then the cells were cultured for 3 days in an incubator at 37 °C, 5% CO_2_. Subsequently, cells were added to 10 μL CCK solution (Dojindo Molecular Tech Inc., Japan), followed by culturing for 2~3 h in an incubator at 37 °C, 5% CO_2_. Cytotoxicity was measured based on the absorbance at 450 nm.

### Culture of patient-derived tumor-like cell clusters (PTCs) from lung cancer

Collected surgically resected tumor tissue and adjacent normal tissue samples were conditioned in ice-cold PBS with 10 mM HEPES and 100 U/mL penicillin-streptomycin (Thermo Fisher Scientific). Tissues were rinsed with ice-cold PBS 6 times. Necrotic areas and adipose tissue were removed as soon as possible. Tissues were minced into small pieces, followed by digesting in digestion solution (5 mL PBS/EDTA 1 mM containing collagenase I, II, and IV (Thermo Fisher Scientific) 200 U/mL each) at 37 °C for 1 h. The digestion was pipetted every 15 min to facilitate cell release, and then collected dissociated cells using 40 mm filters (BD Falcon). After 10 min centrifugation (300 × 3 g, 4 °C), cell pellets were resuspended in a PTC growth medium and seeded in a low-attachment-surface dish at a concentration of 10^5^ cells/cm^2^. Subsequently, cells were cultured in an incubator at 37 °C, 5% CO_2_.

### PTC drug assay

PTCs that were more than 40 mm in diameter were collected using 40 mm filters (BD Falcon), centrifuged at 300 × *g* at 4 °C for 10 min, rinsed with ice-cold PBS, followed by resuspending with G/I-PTC growth medium. 100 μL of a medium containing 30–50 PTCs was seeded into a Teflon-modified chip (GeneX Health, GX-01), then 50 μL of PTC growth medium containing the ADC drug was added to the well, and the plates were incubated at 37 °C, 5% CO_2_. Images of each well were screened with the Nikon Ti-U microscope system. The drug response was evaluated by the ratio of change in area of PTCs: area of PTC after treatment/before treatment.

### xenografted tumor model in mice

Four million NCI-H520 cells mixed in Matrigel (Corning) were injected into one flank of the 6 mice to generate xenograft tumors. When the tumor volumes reached approximately 50–80 mm^3^, we then randomly assigned them to the different treatment arms. Then, the ADC drugs (10 mg/kg) with a total volume of 100 mL per treatment were administered through the tail vein. Treatments were carried out once every week; during this time, tumors were measured with calipers once per week, and body weights were recorded. Mice were sedated with isoflurane before measurements and treatments. Tumor volumes were calculated by the formula (length × width^2^/2) as described previously [47]. The xenografted tumor models were also established with NCI-H226, SK-MES-1, and PC9 cell lines, and were performed in the same methods as described above. In the NCI-H226 and SK-MES-1 xenograft models, different doses (1 mg/kg, 3 mg/kg, 10 mg/kg) or dosing frequencies (QW or Q3W) were tested for efficacy assessment, with other steps unchanged from above.

### Quantification and statistical analysis

A student’s *t*-test was used to calculate differences in tumor volumes between the two groups. For comparisons between multiple groups, a one-way ANOVA was used with a Bonferroni post-test. For Kaplan–Meier survival analysis, a Log-rank (Mantel-Cox) test was used to compare each of the arms. Differences between the two groups were presented as the mean ± SD, as noted in the Figure Legends. Experimental sample numbers (*n*) are indicated in the Figure Legends. For all analyses, statistical significance was defined as *p* < 0.05; all statistical analysis was performed with GraphPad Prism 9.0.

## Supplementary information

Supplemental Table 1

Supplemental Table 2

Supplemental Material

original data

## Data Availability

All data needed to evaluate the conclusions in the paper are present in the paper and/or the Supplementary Information. Related information will be provided upon reasonable request.
