# Supplementary material for: Development of antibody drug conjugates targeting epithelial membrane protein 2-highly expressed lung cancer
Source: Cell Death Dis. 2025 Oct 31;16(1):771. doi: 10.1038/s41419-025-08125-7 (PMC12579237; doi:10.1038/s41419-025-08125-7)
Supplement: Supplementary file 1 — Supplemental Table 1 [file 41419_2025_8125_MOESM1_ESM.docx]

**Supplemental Table1**

| Antibody | MFI | Positive (%) | The ratio of antibody to IgG MFI |
| --- | --- | --- | --- |
| CL007268 | 15452 | 74% | 7.75 |
| CL005091 | 5485 | 37% | 2.75 |
| CL036616 | 6276 | 42% | 3.15 |
| CL004692 | 15979 | 81% | 8.01 |
| CL028351 | 5974 | 39% | 3.00 |
| CL024721 | 5664 | 40% | 2.84 |
| CL024015 | 35284 | 96% | 17.70 |
| CL027482 | 12866.5 | 69% | 6.45 |
| CL036620 | 4536.5 | 32% | 2.28 |
| CL022433 | 5347.5 | 37% | 2.68 |
| CL041369 | 5175 | 33% | 2.60 |
| CL026939 | 6938.5 | 47% | 3.48 |
| CL048461 | 3257 | 19% | 1.63 |
| CL049067 | 3817.5 | 25% | 1.91 |
| CL048962 | 6037.5 | 42% | 3.03 |
| CL046737 | 3705 | 24% | 1.86 |
| CL049071 | 3819.5 | 22% | 1.92 |
| CL029385 | 5740 | 39% | 2.88 |
| CL049596 | 3781.5 | 25% | 1.90 |
| CL039708 | 5260.5 | 30% | 2.64 |
| CL026162 | 5819 | 43% | 2.92 |
| CL032864 | 3598 | 23% | 1.80 |
| CL034616 | 4246.5 | 31% | 2.13 |
| CL034741 | 6660.5 | 43% | 3.34 |
| CL047535 | 2280 | 7% | 1.14 |
| CL037170 | 2588 | 12% | 1.30 |
| CL044826 | 6899 | 44% | 3.46 |
| CL027470 | 11152 | 64% | 5.59 |
| CL028501 | 4094 | 29% | 2.05 |
| CL028506 | 7745 | 50% | 3.88 |
| CL038197 | 4815 | 34% | 2.41 |
| CL029550 | 7333 | 49% | 3.68 |
| CL031024 | 9415 | 58% | 4.72 |
| CL037570 | 3048 | 17% | 1.53 |
| CL041161 | 3107 | 17% | 1.56 |
| CL047312 | 8361 | 54% | 4.19 |
| CL049944 | 3128 | 18% | 1.57 |
| CL048862 | 4009 | 27% | 2.01 |
| CL049133 | 3315.5 | 17% | 1.66 |
| CL049110 | 2866 | 18% | 1.44 |
| CL049216 | 2528.5 | 13% | 1.27 |
| CL048133 | 3589 | 23% | 1.80 |
| CL049127 | 16801.5 | 81% | 8.43 |
| CL039103 | 4354 | 30% | 2.18 |
| CL043320 | 4105 | 30% | 2.06 |
| CL045257 | 4828.5 | 33% | 2.42 |
| CL028960 | 12632 | 68% | 6.34 |
| CL028011 | 3948 | 26% | 1.98 |
| CL028004 | 2880 | 15% | 1.44 |
| CL030182 | 4441 | 28% | 2.23 |
| CL029875 | 13798 | 66% | 6.92 |
| CL029248 | 3570 | 23% | 1.79 |
| CL032511 | 4450 | 27% | 2.23 |
| CL032586 | 4668.5 | 32% | 2.34 |
| CL032581 | 3394 | 18% | 1.70 |
| CL031023 | 4867.5 | 34% | 2.44 |
| CL032857 | 6477 | 45% | 3.25 |
| CL044165 | 3615 | 26% | 1.81 |
| CL033249 | 2903 | 17% | 1.46 |
| CL033251 | 21003.5 | 75% | 10.53 |
| CL033701 | 4667 | 31% | 2.34 |
| CL034122 | 3527.5 | 24% | 1.77 |
| CL034304 | 3014 | 17% | 1.51 |
| CL044406 | 2458.5 | 12% | 1.23 |
| CL046251 | 2099 | 11% | 1.05 |
| CL042513 | 4025.5 | 28% | 2.02 |
| CL028857 | 11413 | 74% | 5.72 |
| CL048421 | 3090 | 18% | 1.55 |
| CL030169 | 30328.5 | 94% | 15.21 |
| CL030062 | 3272.5 | 21% | 1.64 |
| CL039486 | 2304.5 | 11% | 1.16 |
| CL012646 | 3644 | 24% | 1.83 |
| CL045042 | 6028 | 40% | 3.02 |
| CL048968 | 3521 | 23% | 1.77 |
| CL028024 | 4104 | 27% | 2.06 |
| CL025711 | 25429 | 79% | 12.75 |
| CL026840 | 6384 | 44% | 3.20 |
| CL002134 | 6411 | 45% | 3.22 |
| CL033837 | 6914 | 46% | 3.47 |
| CL032881 | 3933.5 | 27% | 1.97 |
| CL042920 | 4139 | 26% | 2.08 |
| CL034104 | 3575 | 25% | 1.79 |
| CL040249 | 3156.5 | 19% | 1.58 |
| CL032141 | 2894.5 | 15% | 1.45 |
| CL029449 | 3120.5 | 18% | 1.56 |
| CL042326 | 8279.5 | 52% | 4.15 |
| CL011177 | 2103 | 8% | 1.05 |
| CL058729 | 3479 | 20% | 1.74 |
| CL026750 | 10936.5 | 67% | 5.48 |
| CL018776 | 6812 | 46% | 3.42 |
| CL042294 | 5388.5 | 41% | 2.70 |
| CL028134 | 3813 | 22% | 1.91 |
| CL025444 | 12018 | 69% | 6.03 |
| CL041108 | 3305.5 | 18% | 1.66 |
| CL026801 | 5573 | 34% | 2.66 |
| CL027825 | 10319 | 57% | 4.93 |
| CL040447 | 3650 | 22% | 1.74 |
| CL031337 | 102248 | 100% | 48.81 |
| CL031224 | 168221 | 85% | 80.30 |
| CL031071 | 8638 | 52% | 4.12 |
| CL035084 | 3706 | 19% | 1.77 |
| CL035116 | 16206 | 79% | 7.74 |
| CL055794 | 5632 | 32% | 2.69 |
| CL024875 | 20523.5 | 88% | 9.80 |
| CL048025 | 4266.5 | 26% | 2.04 |
| CL048128 | 5677 | 35% | 2.71 |
| CL006767 | 5382.5 | 36% | 2.57 |
| CL043799 | 6644.5 | 41% | 3.17 |
| CL031757 | 5395 | 32% | 2.58 |
| CL025870 | 12571 | 67% | 6.00 |
| CL002523 | 18056 | 82% | 8.62 |
| CL033912 | 7980.5 | 49% | 3.81 |
| CL021124 | 4901.5 | 35% | 2.34 |
| CL021676 | 3695 | 26% | 1.76 |
| CL021989 | 8616.5 | 52% | 4.11 |
| CL015453 | 4112 | 32% | 1.96 |
| CL038747 | 3574.5 | 21% | 1.71 |
| CL027365 | 9724 | 57% | 4.64 |
| CL029339 | 8453.5 | 51% | 4.04 |
| CL029065 | 83930 | 99% | 40.06 |
| CL029334 | 7328 | 46% | 3.50 |
| CL028844 | 5182 | 43% | 2.47 |
| CL029603 | 5932.5 | 37% | 2.83 |
| CL028868 | 10129 | 57% | 4.83 |
| CL005252 | 6820 | 44% | 3.26 |
| CL033363 | 4043 | 25% | 1.93 |
| CL028758 | 3728 | 24% | 1.78 |
| CL017797 | 4858.5 | 31% | 2.32 |
| CL012212 | 15944.5 | 72% | 7.61 |
| CL026724 | 14464 | 71% | 6.90 |
| CL045144 | 50585.5 | 99% | 24.15 |
| CL035826 | 4569.5 | 31% | 2.18 |
| CL028247 | 18360 | 81% | 8.76 |
| CL039571 | 6526.5 | 41% | 3.12 |
| CL034859 | 49407 | 96% | 23.58 |
| CL002344 | 7665.5 | 47% | 3.66 |
| CL002415 | 13226.5 | 69% | 6.31 |
| CL033745 | 12744 | 63% | 6.08 |
| CL036063 | 7206 | 46% | 3.44 |
| CL003569 | 22861.5 | 75% | 10.91 |
| CL004953 | 6161 | 39% | 2.94 |
| CL047277 | 4147.5 | 22% | 1.98 |
| CL037871 | 4176 | 23% | 1.99 |
| CL048223 | 3353 | 17% | 1.60 |
| CL032725 | 23564.5 | 87% | 11.25 |
| CL009734 | 4136.5 | 32% | 1.97 |
| CL049814 | 7662.5 | 48% | 3.66 |
| CL035114 | 7017.5 | 45% | 3.35 |
| CL036448 | 4388 | 27% | 2.09 |
| CL012909 | 5324 | 38% | 2.54 |
| CL014133 | 5861 | 41% | 2.80 |
| CL032389 | 5908.5 | 37% | 2.82 |
| CL032787 | 11182.5 | 61% | 5.34 |
| CL032425 | 22556 | 84% | 10.77 |
| CL015576 | 4747.5 | 35% | 2.27 |
| CL015911 | 4016 | 28% | 1.92 |
| CL032821 | 2900.5 | 15% | 1.38 |
| CL033366 | 5957 | 37% | 2.84 |
| CL031116 | 4718 | 30% | 2.25 |
| CL034158 | 4048 | 23% | 1.93 |
| CL013686 | 6095 | 41% | 2.91 |
| CL031444 | 12943.5 | 65% | 6.18 |
| CL019793 | 7412 | 46% | 3.54 |
| CL020925 | 6414 | 45% | 3.06 |
| CL027855 | 8066.5 | 50% | 3.85 |
| CL028295 | 15297 | 71% | 7.30 |
| CL029365 | 7515.5 | 47% | 3.59 |
| CL028635 | 5739 | 39% | 2.74 |
| CL029277 | 6501.5 | 40% | 3.10 |
| CL018146 | 10992 | 59% | 5.25 |
| CL034563 | 21576.5 | 84% | 10.30 |
| CL035071 | 49816.5 | 92% | 23.78 |
| CL021074 | 3983 | 30% | 1.90 |
| CL019334 | 4425 | 31% | 2.11 |
| CL031952 | 9503.5 | 55% | 4.54 |
| CL022000 | 6159 | 40% | 2.94 |
| CL015051 | 4892.5 | 33% | 2.34 |
| CL018104 | 7516 | 48% | 3.59 |
| CL023781 | 5036 | 35% | 2.40 |
| CL017350 | 13435.5 | 79% | 6.41 |
| CL022681 | 3804 | 28% | 1.82 |
| CL027933 | 10039 | 56% | 4.79 |
| CL024959 | 3550.5 | 22% | 1.69 |
| CL026689 | 14407.5 | 72% | 6.88 |
| CL024401 | 5367 | 35% | 2.56 |
| CL037054 | 3527.5 | 20% | 1.68 |
| CL034768 | 9037 | 54% | 4.31 |
| CL027582 | 19166.5 | 79% | 9.15 |
| CL026542 | 7854 | 44% | 3.25 |
| CL019468 | 4380.5 | 23% | 1.81 |
| CL042730 | 4113 | 21% | 1.70 |
| CL001237 | 4040.5 | 20% | 1.67 |
| CL028961 | 5575.5 | 27% | 2.30 |
| CL028256 | 16079 | 73% | 6.65 |
| CL025059 | 9635 | 52% | 3.98 |
| CL028715 | 4984 | 27% | 2.06 |
| CL028578 | 4428.5 | 22% | 1.83 |
| CL047472 | 2801.5 | 13% | 1.16 |
| CL030613 | 9914.5 | 52% | 4.10 |
| CL029867 | 10538 | 55% | 4.36 |
| CL030578 | 13374.5 | 63% | 5.53 |
| CL026859 | 4798.5 | 30% | 1.98 |
| CL029297 | 3978.5 | 25% | 1.64 |
| CL031395 | 4570 | 24% | 1.89 |
| CL028120 | 3630 | 20% | 1.50 |
| CL024002 | 6280 | 33% | 2.60 |
| CL026203 | 7054 | 41% | 2.92 |
| CL030572 | 7979.5 | 43% | 3.30 |
| CL011665 | 7738.5 | 45% | 3.20 |
| CL034784 | 5149 | 30% | 2.13 |
| CL030550 | 4585.5 | 25% | 1.90 |
| CL041477 | 4974 | 21% | 2.06 |
| CL037947 | 7439 | 42% | 3.07 |
| CL027282 | 8278.5 | 46% | 3.42 |
| CL042665 | 3416 | 14% | 1.41 |
| CL045975 | 3834.5 | 20% | 1.58 |
| CL039603 | 4366.5 | 24% | 1.80 |
| CL048686 | 5876 | 33% | 2.43 |
| CL044678 | 4577 | 24% | 1.89 |
| CL009223 | 4477.5 | 28% | 1.85 |
| CL032823 | 4212 | 24% | 1.74 |
| CL002248 | 6292 | 35% | 2.60 |
| CL061977 | 5236.5 | 28% | 2.16 |
| CL010906 | 10872.5 | 59% | 4.49 |
| CL008838 | 6491 | 41% | 2.68 |
| CL006369 | 9489.5 | 51% | 3.92 |
| CL031376 | 6328 | 35% | 2.62 |
| CL032638 | 4365.5 | 21% | 1.80 |
| CL028661 | 7448 | 44% | 3.08 |
| CL009510 | 8207.5 | 46% | 3.39 |
| CL048287 | 4964.5 | 26% | 2.05 |
| CL040809 | 5358 | 29% | 2.21 |
| CL000815 | 3626 | 16% | 1.50 |
| CL030960 | 3215 | 15% | 1.33 |
| CL048362 | 4196.5 | 21% | 1.73 |
| CL043244 | 4371 | 17% | 1.81 |
| CL040218 | 5135 | 25% | 2.12 |
| CL039998 | 4245 | 22% | 1.75 |
| CL054385 | 4574.5 | 23% | 1.89 |
| CL041708 | 26327.5 | 75% | 10.88 |
| CL042194 | 7131.5 | 41% | 2.95 |
| CL039604 | 4200 | 25% | 1.74 |
| CL006089 | 21035.5 | 77% | 8.69 |
| CL019487 | 3799 | 28% | 1.57 |
| CL027474 | 4897.5 | 31% | 2.02 |
| CL029572 | 3155 | 12% | 1.30 |
| CL034337 | 2907.5 | 12% | 1.20 |
| CL036124 | 4126 | 20% | 1.71 |
| CL017416 | 7780 | 43% | 3.22 |
| CL041602 | 4007 | 19% | 1.66 |
| CL049135 | 2651.5 | 8% | 1.10 |
| CL008933 | 13861 | 62% | 5.73 |
| CL003903 | 6907.5 | 39% | 2.85 |
| CL002987 | 9702 | 51% | 4.01 |
| CL013049 | 4089 | 22% | 1.69 |
| CL024639 | 5190.5 | 38% | 2.15 |
| CL031607 | 4624 | 24% | 1.91 |
| CL005195 | 5341.5 | 35% | 2.21 |
| CL053547 | 5812 | 32% | 2.40 |
| CL003967 | 5935.5 | 34% | 2.45 |
| CL053643 | 3161 | 12% | 1.31 |
| CL053463 | 5385 | 22% | 2.23 |
| CL053788 | 3787.5 | 18% | 1.57 |
| CL034959 | 4538.5 | 22% | 1.88 |
| CL046913 | 3330 | 14% | 1.38 |
| CL053431 | 4291.5 | 24% | 1.77 |
| CL048117 | 3226 | 14% | 1.33 |
| CL025863 | 5006.5 | 27% | 2.07 |
| CL036767 | 3759.5 | 16% | 1.55 |
| CL037868 | 15751.5 | 74% | 6.51 |
| CL053609 | 3223 | 25% | 1.33 |
| CL026482 | 16195.5 | 66% | 6.69 |
| CL043578 | 20064.5 | 82% | 8.29 |
| CL024907 | 8174.5 | 45% | 3.38 |
| CL046789 | 3676.5 | 14% | 1.52 |
| CL053522 | 2542 | 8% | 1.05 |
| CL046513 | 2822.5 | 11% | 1.17 |
| CL032765 | 14756 | 65% | 6.10 |
| CL036605 | 6784 | 37% | 2.80 |
| CL044234 | 4239 | 21% | 1.75 |
| CL011742 | 6961.5 | 37% | 2.88 |
| CL019296 | 2501 | 8% | 1.03 |
| CL039697 | 3800.5 | 26% | 2.26 |
| CL005245 | 7375.5 | 45% | 4.39 |
| CL037232 | 4290.5 | 30% | 2.55 |
| CL031887 | 3060 | 20% | 1.82 |
| CL004805 | 3644.5 | 27% | 2.17 |
| CL026913 | 9583 | 57% | 5.70 |
| CL031460 | 4434 | 33% | 2.64 |
| CL009825 | 5094 | 33% | 3.03 |
| CL044652 | 3477.5 | 20% | 2.07 |
| CL007525 | 112288 | 99% | 66.84 |
| CL023914 | 2463 | 14% | 1.47 |
| CL028841 | 32211 | 95% | 19.17 |
| CL004981 | 5832 | 40% | 3.47 |
| CL038099 | 19400 | 92% | 11.55 |
| CL031602 | 7332 | 46% | 4.36 |
| CL034705 | 7572 | 47% | 4.51 |
| CL037306 | 3026 | 17% | 1.80 |
| CL013011 | 2479 | 19% | 1.48 |
| CL042037 | 3998 | 30% | 2.38 |
| CL029834 | 4937.5 | 33% | 2.94 |
| CL038316 | 9257 | 54% | 5.51 |
| CL032124 | 3508.5 | 27% | 2.09 |
| CL053645 | 3209 | 24% | 1.91 |
| CL012140 | 17243 | 86% | 10.26 |
| CL054434 | 2694 | 15% | 1.60 |
| CL028638 | 4013 | 26% | 2.39 |
| CL036946 | 2776.5 | 20% | 1.65 |
| CL021085 | 3449.5 | 26% | 2.05 |
| CL032076 | 5422 | 37% | 3.23 |
| CL028112 | 1896.5 | 10% | 1.13 |
| CL053552 | 3305.5 | 22% | 1.97 |
| CL032054 | 2798 | 21% | 1.67 |
| CL032021 | 4641 | 29% | 2.76 |
| CL027600 | 5826 | 37% | 3.47 |
| CL036564 | 4600.5 | 29% | 2.74 |
| CL044923 | 3576 | 24% | 2.13 |
| CL021525 | 15064 | 83% | 8.97 |
| CL037402 | 7181.5 | 46% | 4.27 |
| CL029583 | 7087.5 | 44% | 4.22 |
| CL031407 | 3068.5 | 22% | 1.83 |
| CL008385 | 5953 | 38% | 3.54 |
| CL018629 | 3268.5 | 22% | 1.95 |
| CL032935 | 3498.5 | 23% | 2.08 |
| CL005612 | 6069 | 42% | 3.61 |
| CL050317 | 4381 | 21% | 2.61 |
| CL005024 | 7301.5 | 44% | 4.35 |
| CL039834 | 3552.5 | 22% | 2.11 |
| CL003519 | 6477.5 | 43% | 3.86 |
| CL003133 | 11033.5 | 66% | 6.57 |
| CL001348 | 3913 | 25% | 2.33 |
| CL044142 | 5781.5 | 31% | 3.44 |
| CL037277 | 3276 | 22% | 1.95 |
| CL028579 | 5231.5 | 34% | 3.11 |
| CL041081 | 5370 | 37% | 3.20 |
| CL024815 | 6145 | 42% | 3.66 |
| CL030421 | 2387 | 14% | 1.42 |
| CL000822 | 4687.5 | 35% | 2.79 |
| CL055906 | 2342 | 12% | 1.39 |
| CL051794 | 2884 | 16% | 1.72 |
| CL057244 | 3036 | 21% | 1.81 |
| CL056857 | 3454 | 16% | 2.06 |
| CL057932 | 4185.5 | 26% | 2.49 |
| CL055620 | 3944 | 28% | 2.35 |
| CL051289 | 3952.5 | 25% | 2.35 |
| CL051079 | 3147 | 20% | 1.87 |
| CL051358 | 48282 | 92% | 28.74 |
| CL055614 | 3828.5 | 28% | 2.28 |
| CL052276 | 4423 | 20% | 2.63 |
| CL054162 | 4855.5 | 36% | 2.89 |
| CL025578 | 5861.5 | 40% | 3.49 |
| CL026390 | 2852 | 17% | 1.70 |
| CL059825 | 2795.5 | 17% | 1.66 |
| CL037757 | 4218 | 28% | 2.51 |
| CL031438 | 3357.5 | 34% | 2.00 |
| CL026351 | 4315 | 29% | 2.57 |
| CL054131 | 3537 | 26% | 2.11 |
| CL024917 | 6960.5 | 45% | 4.14 |
| CL051499 | 4962.5 | 36% | 2.95 |
| CL054763 | 3611 | 26% | 2.15 |
| CL044847 | 2915.5 | 16% | 1.74 |
| CL055635 | 3820 | 24% | 2.27 |
| CL055978 | 4742 | 34% | 2.82 |
| CL041796 | 6387 | 41% | 3.80 |
| CL007826 | 5010 | 36% | 2.98 |
| CL039468 | 5158 | 30% | 3.07 |
| CL048699 | 3442 | 24% | 2.05 |
| CL055867 | 6337 | 36% | 3.77 |
| CL057132 | 5016.5 | 32% | 2.99 |
| CL000600 | 4013 | 26% | 2.39 |
| CL051469 | 5219 | 39% | 3.11 |
| CL051759 | 6975 | 44% | 4.15 |
| CL050831 | 16372.5 | 85% | 9.75 |
| CL052218 | 6611.5 | 41% | 3.94 |
| CL059838 | 3067 | 18% | 1.83 |
| CL025681 | 11925 | 71% | 7.05 |
| CL027769 | 4571.5 | 33% | 2.70 |
| CL027904 | 5216 | 37% | 3.08 |
| CL029825 | 4134.5 | 30% | 2.44 |
| CL053171 | 4289 | 35% | 2.54 |
| CL055690 | 6454 | 47% | 3.82 |
| CL055847 | 2466 | 18% | 1.46 |
| CL059906 | 2741 | 22% | 1.62 |
| CL057114 | 3860 | 32% | 2.28 |
| CL029496 | 7795.5 | 54% | 4.61 |
| CL036111 | 5457.5 | 42% | 3.23 |
| CL001759 | 6410 | 45% | 3.79 |
| CL033957 | 784703 | 97% | 463.91 |
| CL022809 | 3230 | 27% | 1.91 |
| CL032035 | 9158.5 | 66% | 5.41 |
| CL054113 | 2987 | 24% | 1.77 |
| CL031384 | 19205.5 | 84% | 11.35 |
| CL028348 | 17836.5 | 89% | 10.54 |
| CL053778 | 3364.5 | 27% | 1.99 |
| CL027501 | 16396.5 | 83% | 9.69 |
| CL039147 | 5703.5 | 43% | 3.37 |
| CL055882 | 11678.5 | 64% | 6.90 |
| CL057814 | 5905 | 44% | 3.49 |
| CL025698 | 11465.5 | 66% | 6.78 |
| CL027015 | 692440 | 94% | 409.36 |
| CL051333 | 6037 | 44% | 3.57 |
| CL024898 | 5019 | 41% | 2.97 |
| CL031935 | 2497 | 19% | 1.48 |
| CL029333 | 7572 | 53% | 4.48 |
| CL032851 | 5611 | 41% | 3.32 |
| CL029400 | 10636 | 73% | 6.29 |
| CL028096 | 3811.5 | 31% | 2.25 |
| CL056880 | 8477 | 57% | 5.01 |
| CL031505 | 22641.5 | 80% | 13.39 |
| CL029298 | 4089 | 34% | 2.42 |
| CL055846 | 7149 | 51% | 4.23 |
| CL057735 | 7542.5 | 53% | 4.46 |
| CL057609 | 9852 | 70% | 5.82 |
| CL004032 | 5850 | 44% | 3.46 |
| CL001530 | 4517 | 35% | 2.67 |
| CL036473 | 7746.5 | 54% | 4.58 |
| CL032125 | 4029 | 37% | 2.38 |
| CL019483 | 5063.5 | 40% | 2.99 |
| CL033294 | 9093 | 59% | 5.38 |
| CL030804 | 14196.5 | 71% | 8.39 |
| CL056740 | 7038 | 50% | 4.16 |
| CL014607 | 10140.5 | 64% | 5.99 |
| CL022074 | 6229 | 47% | 3.68 |
| CL054026 | 4635.5 | 33% | 2.74 |
| CL033991 | 3054 | 26% | 1.81 |
| CL000748 | 4661 | 38% | 2.76 |
| CL000616 | 4945.5 | 36% | 2.92 |
| CL031077 | 7631.5 | 53% | 4.51 |
| CL005228 | 5603.5 | 43% | 3.31 |
| CL008822 | 6680.5 | 49% | 3.95 |
| CL000068 | 4327.5 | 35% | 2.56 |
| CL049516 | 7918 | 54% | 4.68 |
| CL029565 | 9721 | 62% | 5.75 |
| CL013872 | 6843 | 49% | 4.05 |
| CL053109 | 3947.5 | 31% | 2.33 |
| CL002918 | 9480 | 60% | 5.60 |
| CL003911 | 15124 | 75% | 8.94 |
| CL027153 | 32050 | 95% | 18.95 |
| CL052165 | 7410.5 | 52% | 4.38 |
| CL027453 | 6241 | 46% | 3.69 |
| CL051926 | 7713 | 55% | 4.56 |
| CL056837 | 5114 | 41% | 3.02 |
| CL055633 | 7683.5 | 54% | 4.54 |
| CL029916 | 6555 | 48% | 3.88 |
| CL024385 | 13776.5 | 75% | 8.14 |
| CL016418 | 23254 | 84% | 13.75 |
| CL013501 | 25954.5 | 85% | 15.34 |
| CL033379 | 8752 | 61% | 5.17 |
| CL005248 | 6654 | 48% | 3.93 |
| CL004655 | 9061.5 | 58% | 5.36 |
| CL004656 | 4746.5 | 37% | 2.81 |
| CL032386 | 14097 | 78% | 8.33 |
| CL006948 | 8353.5 | 56% | 4.94 |
| CL002007 | 3673 | 31% | 2.17 |
| CL004822 | 12980 | 67% | 7.67 |
| CL004928 | 4927 | 39% | 2.91 |
| CL004853 | 6120.5 | 46% | 3.62 |
| CL037114 | 5117.5 | 40% | 3.03 |
| CL059694 | 3511.5 | 27% | 2.08 |
| CL007901 | 3890.5 | 32% | 2.30 |
| CL015665 | 7983 | 56% | 4.72 |
| CL021216 | 7588 | 53% | 4.49 |
| CL037345 | 2941.5 | 21% | 1.74 |
| CL024278 | 6708.5 | 49% | 3.97 |
| CL037918 | 6084.5 | 45% | 3.60 |
| CL055634 | 6053 | 44% | 3.58 |
| CL007194 | 6293.5 | 47% | 3.72 |
| CL007473 | 9633.5 | 60% | 5.70 |
| CL036331 | 9908.5 | 61% | 5.86 |
| CL018065 | 2404 | 19% | 1.51 |
| CL061241 | 4653.5 | 45% | 2.92 |
| CL035176 | 2289 | 16% | 1.44 |
| CL044008 | 3665 | 35% | 2.30 |
| CL028107 | 4561 | 44% | 2.86 |
| CL027975 | 13486 | 80% | 8.47 |
| CL037876 | 3632 | 38% | 2.28 |
| CL055314 | 3925 | 38% | 2.47 |
| CL007571 | 8006 | 72% | 5.03 |
| CL031816 | 2930 | 31% | 1.84 |
| CL034491 | 8193 | 73% | 5.15 |
| CL007814 | 1966.5 | 18% | 1.24 |
| CL007724 | 1920.5 | 19% | 1.21 |
| CL049718 | 2637 | 24% | 1.66 |
| CL049461 | 11902.5 | 75% | 7.48 |
| CL003791 | 5949 | 56% | 3.74 |
| CL040212 | 6783.5 | 66% | 4.26 |
| CL034892 | 8816 | 67% | 5.54 |
| CL029847 | 5772 | 59% | 3.63 |
| CL058378 | 2493 | 23% | 1.57 |
| CL005986 | 4183.5 | 40% | 2.63 |
| CL030323 | 7600.5 | 63% | 4.77 |
| CL009182 | 4986 | 48% | 3.13 |
| CL055687 | 31197 | 93% | 19.60 |
| CL058789 | 26127 | 92% | 16.41 |
| CL009314 | 21403 | 98% | 13.44 |
| CL056150 | 2876 | 28% | 1.81 |
| CL014777 | 8247 | 67% | 5.18 |
| CL050777 | 2847 | 29% | 1.79 |
| CL060277 | 4233.5 | 42% | 2.66 |
| CL054803 | 5624.5 | 54% | 3.53 |
| CL049215 | 2097.5 | 16% | 1.32 |
| CL030407 | 4813.5 | 47% | 3.02 |
| CL011784 | 1815 | 16% | 1.14 |
| CL033006 | 3487 | 37% | 2.19 |
| CL002325 | 7183 | 65% | 4.51 |
| CL010157 | 4235 | 43% | 2.66 |
| CL023056 | 1987 | 15% | 1.25 |
| CL024414 | 4835 | 47% | 3.04 |
| CL057398 | 3971 | 38% | 2.49 |
| CL032479 | 2437.5 | 23% | 1.53 |
| CL033308 | 26190 | 92% | 16.45 |
| CL053505 | 3756 | 33% | 2.36 |
| CL030197 | 9210 | 76% | 5.79 |
| CL005511 | 5891 | 55% | 3.70 |
| CL005468 | 5739 | 52% | 3.60 |
| CL047889 | 3471 | 35% | 2.18 |
| CL056008 | 2652 | 25% | 1.67 |
| CL026780 | 3934 | 39% | 2.47 |
| CL041683 | 5464 | 52% | 3.43 |
| CL019581 | 27973 | 92% | 17.57 |
| CL054892 | 3727 | 36% | 2.34 |
| CL017124 | 3601 | 41% | 2.26 |
| CL011184 | 3976 | 38% | 2.50 |
| CL026990 | 19716 | 92% | 12.38 |
| CL007893 | 6016.5 | 56% | 3.78 |
| CL020905 | 22238 | 92% | 13.97 |
| CL049024 | 5922.5 | 54% | 3.72 |
| CL036618 | 4515 | 45% | 2.84 |
| CL008783 | 4667 | 46% | 2.93 |
| CL053411 | 4048 | 39% | 2.54 |
| CL005826 | 3010.5 | 25% | 1.89 |
| CL032653 | 2668 | 26% | 1.68 |
| CL031241 | 19707 | 91% | 12.38 |
| CL036013 | 2623 | 24% | 1.65 |
| CL047799 | 4695 | 46% | 2.95 |
| CL035904 | 3217 | 34% | 2.02 |
| CL032148 | 4570 | 46% | 2.87 |
| CL037471 | 2600.5 | 24% | 1.63 |
| CL054802 | 2161 | 20% | 1.36 |
| CL031661 | 2336.5 | 22% | 1.47 |
| CL007368 | 2974 | 27% | 1.87 |
| CL005737 | 4760 | 47% | 2.99 |
| CL002203 | 6647 | 59% | 4.18 |
| CL003310 | 9214 | 70% | 5.79 |
| CL013841 | 3859 | 40% | 2.42 |
| CL006206 | 5607 | 52% | 3.52 |
| CL005016 | 6156 | 56% | 3.87 |
| CL003150 | 4309 | 43% | 2.71 |
| CL002247 | 10334 | 79% | 6.49 |
| CL002658 | 10184 | 83% | 6.40 |
| CL025585 | 17583 | 97% | 11.04 |
| CL059930 | 1897 | 14% | 1.19 |
| CL053213 | 3993 | 42% | 2.51 |
| CL032327 | 2455 | 21% | 1.54 |
| CL026726 | 4546 | 45% | 2.86 |
| CL017048 | 3660 | 40% | 2.30 |
| CL031583 | 7567.5 | 63% | 4.75 |
| CL004601 | 4463 | 45% | 2.80 |
| CL008177 | 3488 | 34% | 2.19 |
| CL009514 | 3518 | 38% | 2.21 |
| CL057119 | 1910 | 12% | 1.20 |
| CL046084 | 4556 | 44% | 2.86 |
| CL019107 | 2101 | 17% | 1.32 |
| CL023500 | 3787.5 | 24% | 1.91 |
| CL006658 | 4994 | 34% | 2.52 |
| CL056096 | 4445.5 | 23% | 2.24 |
| CL023868 | 4181 | 29% | 2.11 |
| CL029874 | 4302.5 | 29% | 2.17 |
| CL024411 | 5390 | 33% | 2.71 |
| CL029856 | 5126 | 34% | 2.58 |
| CL025051 | 12313 | 80% | 6.20 |
| CL039330 | 7725 | 51% | 3.89 |
| CL014682 | 3625 | 22% | 1.83 |
| CL047818 | 3443.5 | 15% | 1.73 |
| CL050546 | 6266 | 41% | 3.16 |
| CL013482 | 3321 | 24% | 1.67 |
| CL055458 | 3696 | 23% | 1.86 |
| CL060148 | 3162 | 13% | 1.59 |
| CL812298 | 2812.5 | 11% | 1.42 |
| CL012881 | 3568 | 18% | 1.80 |
| CL008005 | 5195 | 37% | 2.62 |
| CL010394 | 7914.5 | 52% | 3.99 |
| CL009926 | 5668.5 | 39% | 2.85 |
| CL009417 | 3708 | 18% | 1.87 |
| CL030559 | 7247 | 48% | 3.65 |
| CL045268 | 3877 | 19% | 1.95 |
| CL026239 | 13699.5 | 75% | 6.90 |
| CL047584 | 3869.5 | 20% | 1.95 |
| CL054217 | 5311 | 40% | 2.67 |
| CL062924 | 3126 | 15% | 1.57 |
| CL019772 | 13107 | 67% | 6.60 |
| CL008398 | 7669.5 | 51% | 3.86 |
| CL061332 | 8647.5 | 57% | 4.36 |
| CL037410 | 11201.5 | 66% | 5.64 |
| CL825915 | 4401.5 | 30% | 2.22 |
| CL059933 | 6447.5 | 44% | 3.25 |
| CL050737 | 22417.5 | 87% | 11.29 |
| CL023441 | 11876 | 67% | 5.98 |
| CL022327 | 11690 | 74% | 5.89 |
| CL058786 | 6402.5 | 42% | 3.22 |
| CL024085 | 3185.5 | 11% | 1.60 |
| CL024087 | 4476 | 28% | 2.25 |
| CL040542 | 7480.5 | 50% | 3.77 |
| CL024162 | 3106 | 18% | 1.56 |
| CL027375 | 11066 | 64% | 5.57 |
| CL056527 | 4353 | 28% | 2.19 |
| CL056441 | 5504 | 34% | 2.77 |
| CL058310 | 4644 | 29% | 2.34 |
| CL051296 | 7008 | 48% | 3.53 |
| CL007727 | 6636 | 44% | 3.34 |
| CL035107 | 6846.5 | 46% | 3.45 |
| CL001026 | 13440.5 | 66% | 6.77 |
| CL027611 | 5006.5 | 30% | 2.52 |
| CL030979 | 7827 | 52% | 3.94 |
| CL030961 | 12662 | 75% | 6.38 |
| CL016308 | 11737.5 | 70% | 5.91 |
| CL035797 | 43584 | 94% | 21.95 |
| CL028935 | 10317 | 58% | 5.20 |
| CL027626 | 3681 | 23% | 1.85 |
| CL031961 | 4897.5 | 29% | 2.47 |
| CL037030 | 23865.5 | 96% | 12.02 |
| CL020865 | 4886.5 | 34% | 2.46 |
| CL022244 | 3084 | 16% | 1.55 |
| CL032426 | 3942 | 26% | 1.99 |
| CL009670 | 7040 | 48% | 3.55 |
| CL027000 | 8857 | 58% | 4.46 |
| CL016365 | 2482 | 7% | 1.25 |
| CL033636 | 4529 | 30% | 2.28 |
| CL050028 | 4702 | 29% | 2.37 |
| CL033342 | 6179.5 | 42% | 3.11 |
| CL034409 | 3947.5 | 24% | 1.99 |
| CL062854 | 3566 | 20% | 1.80 |
| CL032089 | 11970 | 69% | 6.03 |
| CL047885 | 12750 | 63% | 6.42 |
| CL033950 | 13255 | 71% | 6.68 |
| CL038090 | 5774 | 41% | 2.91 |
| CL060313 | 14476.5 | 73% | 7.29 |
| CL058084 | 3248 | 16% | 1.64 |
| CL019331 | 6849.5 | 47% | 3.45 |
| CL056152 | 5326.5 | 33% | 2.68 |
| CL025899 | 12579.5 | 69% | 6.34 |
| CL051795 | 7481 | 50% | 3.77 |
| CL019384 | 17581.5 | 77% | 8.85 |
| CL052088 | 6948 | 47% | 3.50 |
| CL020040 | 8737.5 | 54% | 4.40 |
| CL015951 | 6292 | 45% | 3.17 |
| CL019598 | 8453 | 55% | 4.26 |
| CL024535 | 3849.5 | 30% | 1.94 |
| CL031396 | 6502 | 44% | 3.27 |
| CL055608 | 4357.5 | 27% | 2.19 |
| CL052503 | 5089.5 | 33% | 2.56 |
| CL019626 | 6934.5 | 48% | 3.49 |
| CL004480 | 6611 | 45% | 3.33 |
| CL038178 | 3264.5 | 17% | 1.64 |
| CL024417 | 5432 | 35% | 2.74 |
| CL022462 | 5285.5 | 35% | 2.66 |
| CL019831 | 5684.5 | 42% | 2.86 |
| CL011291 | 5115.5 | 18% | 2.13 |
| CL060207 | 4199 | 18% | 1.75 |
| CL016849 | 4204.5 | 16% | 1.75 |
| CL060116 | 5363.5 | 20% | 2.24 |
| CL013595 | 17051 | 80% | 7.11 |
| CL036824 | 7407 | 37% | 3.09 |
| CL029696 | 4231.5 | 17% | 1.76 |
| CL006317 | 11200 | 52% | 4.67 |
| CL021250 | 9184 | 47% | 3.83 |
| CL026564 | 7565.5 | 37% | 3.15 |
| CL060912 | 6188 | 29% | 2.58 |
| CL028533 | 7760 | 39% | 3.23 |
| CL060409 | 7058 | 34% | 2.94 |
| CL060117 | 29197 | 86% | 12.17 |
| CL029208 | 7476 | 42% | 3.12 |
| CL029473 | 8071 | 39% | 3.36 |
| CL031932 | 3798.5 | 16% | 1.58 |
| CL021121 | 3794.5 | 22% | 1.58 |
| CL004444 | 10718.5 | 52% | 4.47 |
| CL032982 | 3070 | 10% | 1.28 |
| CL035511 | 7900.5 | 39% | 3.29 |
| CL033354 | 6786.5 | 35% | 2.83 |
| CL020922 | 8949 | 45% | 3.73 |
| CL050044 | 2881 | 7% | 1.20 |
| CL031873 | 5013.5 | 18% | 2.09 |
| CL042887 | 3809 | 13% | 1.59 |
| CL036050 | 7887.5 | 40% | 3.29 |
| CL043234 | 10519.5 | 51% | 4.38 |
| CL024360 | 5569.5 | 31% | 2.32 |
| CL052301 | 9959 | 49% | 4.15 |
| CL055223 | 3628.5 | 13% | 1.51 |
| CL018601 | 4328.5 | 12% | 1.80 |
| CL010055 | 15459 | 68% | 6.44 |
| CL039532 | 7852.5 | 41% | 3.27 |
| CL019207 | 4903.5 | 22% | 2.04 |
| CL033749 | 26240.5 | 82% | 10.94 |
| CL044456 | 4814.5 | 18% | 2.01 |
| CL043942 | 8198 | 43% | 3.42 |
| CL007944 | 11794.5 | 54% | 4.92 |
| CL025360 | 11690.5 | 58% | 4.87 |
| CL019769 | 11423 | 54% | 4.76 |
| CL017886 | 7093.5 | 36% | 2.96 |
| CL043064 | 8181 | 43% | 3.41 |
| CL012477 | 3685 | 19% | 1.54 |
| CL053089 | 10800 | 52% | 4.50 |
| CL023971 | 10217 | 50% | 4.26 |
| CL062800 | 22290 | 73% | 9.29 |
| CL033716 | 7884 | 40% | 3.29 |
| CL018525 | 3735 | 14% | 1.56 |
| CL019730 | 4122.5 | 22% | 1.72 |
| CL036789 | 6964.5 | 34% | 2.90 |
| CL017471 | 4959 | 28% | 2.07 |
| CL016087 | 3270 | 11% | 1.36 |
| CL004788 | 6780 | 37% | 2.83 |
| CL058110 | 4447.5 | 18% | 1.85 |
| CL825624 | 3715 | 10% | 1.55 |
| CL014968 | 5520.5 | 26% | 2.30 |
| CL049569 | 3199.5 | 10% | 1.33 |
| CL058673 | 3407.5 | 10% | 1.42 |
| CL059576 | 5681.5 | 26% | 2.37 |
| CL063061 | 3280 | 7% | 1.37 |
| CL019136 | 6786 | 38% | 2.83 |
| CL046958 | 4702 | 19% | 1.96 |
| CL060914 | 5786 | 27% | 2.41 |
| CL011375 | 3940.5 | 22% | 1.64 |
| CL823714 | 3153 | 9% | 1.31 |
| CL815218 | 2235 | 1% | 0.93 |
| CL057293 | 4495 | 18% | 1.87 |
| CL061984 | 3719.5 | 17% | 1.55 |
| CL061432 | 4548.5 | 16% | 1.90 |
| CL040555 | 5160 | 25% | 2.15 |
| CL062286 | 2936 | 8% | 1.22 |
| CL054278 | 2844.5 | 11% | 1.19 |
| CL015384 | 9718.5 | 48% | 4.05 |
| CL051465 | 6844.5 | 30% | 2.85 |
| CL046309 | 5853.5 | 28% | 2.44 |
| CL014681 | 2697 | 9% | 1.12 |
| CL022208 | 6401 | 30% | 2.67 |
| CL023710 | 10771.5 | 52% | 4.49 |
| CL056140 | 6976 | 32% | 2.91 |
| CL062911 | 4213.5 | 16% | 1.76 |
| CL042640 | 3177 | 9% | 1.32 |
| CL035347 | 3929 | 13% | 1.64 |
| CL031852 | 4616.5 | 19% | 1.92 |
| CL027103 | 11705.5 | 55% | 4.88 |
| CL017073 | 5672 | 23% | 2.36 |
| CL002438 | 4733.5 | 16% | 1.97 |
| CL028874 | 4796 | 18% | 2.00 |
| CL027313 | 3535.5 | 11% | 1.47 |
| CL042880 | 5168.5 | 26% | 2.15 |
| CL032545 | 11230.5 | 52% | 4.68 |
| CL053046 | 3798.5 | 12% | 1.58 |
| CL012671 | 9058.5 | 47% | 3.78 |
| CL012687 | 3599.5 | 14% | 1.50 |
| CL054334 | 3855.5 | 22% | 1.98 |
| CL031416 | 10861.5 | 70% | 5.57 |
| CL063307 | 3645 | 22% | 1.87 |
| CL005652 | 4068.5 | 24% | 2.09 |
| CL022240 | 4205 | 28% | 2.16 |
| CL039082 | 6190.5 | 43% | 3.18 |
| CL053555 | 5426 | 35% | 2.78 |
| CL057423 | 3455 | 20% | 1.77 |
| CL053621 | 3654.5 | 24% | 1.88 |
| CL021091 | 5106 | 37% | 2.62 |
| CL013322 | 6483.5 | 46% | 3.33 |
| CL026147 | 9418.5 | 58% | 4.83 |
| CL023744 | 2925.5 | 14% | 1.50 |
| CL057424 | 4886.5 | 35% | 2.51 |
| CL027311 | 6692 | 45% | 3.43 |
| CL030444 | 3201.5 | 14% | 1.64 |
| CL054651 | 3375.5 | 17% | 1.73 |
| CL027213 | 20454 | 94% | 10.50 |
| CL036944 | 8345.5 | 55% | 4.28 |
| CL023132 | 7739 | 52% | 3.97 |
| CL022942 | 4237 | 29% | 2.17 |
| CL022748 | 5134 | 37% | 2.63 |
| CL058832 | 3886 | 25% | 1.99 |
| CL030471 | 6738.5 | 47% | 3.46 |
| CL025410 | 5494 | 40% | 2.82 |
| CL025400 | 3872.5 | 25% | 1.99 |
| CL023814 | 3692 | 27% | 1.89 |
| CL002374 | 4952 | 35% | 2.54 |
| CL021554 | 7264 | 50% | 3.73 |
| CL055285 | 3947 | 26% | 2.03 |
| CL027878 | 7276.5 | 50% | 3.73 |
| CL051598 | 4430 | 29% | 2.27 |
| CL055657 | 6388.5 | 45% | 3.28 |
| CL021483 | 4054 | 26% | 2.08 |
| CL017663 | 8479 | 54% | 4.35 |
| CL017548 | 4365.5 | 33% | 2.24 |
| CL055120 | 4184 | 27% | 2.15 |
| CL013094 | 6927 | 47% | 3.56 |
| CL035619 | 4903.5 | 34% | 2.52 |
| CL022545 | 6445 | 46% | 3.31 |
| CL055526 | 3407.5 | 19% | 1.75 |
| CL011949 | 7372 | 50% | 3.78 |
| CL030595 | 5357 | 39% | 2.75 |
| CL014848 | 5087.5 | 38% | 2.61 |
| CL013171 | 8474.5 | 54% | 4.35 |
| CL014802 | 6628 | 45% | 3.40 |
| CL045599 | 5438.5 | 42% | 2.79 |
| CL023662 | 7865 | 54% | 4.04 |
| CL060889 | 7090.5 | 48% | 3.64 |
| CL053414 | 3099 | 17% | 1.59 |
| CL055201 | 6827.5 | 47% | 3.50 |
| CL021082 | 5775 | 39% | 2.96 |
| CL018294 | 2939.5 | 14% | 1.51 |
| CL029903 | 4153 | 26% | 2.13 |
| CL045587 | 3150.5 | 13% | 1.62 |
| CL008326 | 16651 | 76% | 8.55 |
| CL055054 | 4479.5 | 31% | 2.30 |
| CL054250 | 11166.5 | 68% | 5.73 |
| CL025381 | 7156 | 49% | 3.67 |
| CL039741 | 3962 | 31% | 2.03 |
| CL055389 | 4111.5 | 22% | 2.11 |
| CL042956 | 4244 | 28% | 2.18 |
| CL050211 | 5071 | 34% | 2.60 |
| CL016703 | 6539.5 | 46% | 3.36 |
| CL021386 | 5523 | 40% | 2.83 |
| CL057876 | 5538.5 | 40% | 2.84 |
| CL063235 | 4395.5 | 28% | 2.26 |
| CL059945 | 3631 | 27% | 1.86 |
| CL058961 | 8434.5 | 55% | 4.33 |
| CL024217 | 7607 | 51% | 3.90 |
| CL023772 | 13691 | 76% | 7.03 |
| CL025370 | 4274.5 | 27% | 2.19 |
| CL050221 | 9425 | 58% | 4.84 |
| CL020273 | 3644 | 19% | 1.87 |
| CL053037 | 6354.5 | 44% | 3.26 |
| CL053562 | 7514.5 | 51% | 3.86 |
| CL009397 | 4495.5 | 27% | 2.31 |
| CL011047 | 7878 | 52% | 4.04 |
| CL060693 | 2999 | 17% | 1.54 |
| CL024248 | 8518 | 56% | 4.37 |
| CL023303 | 5119 | 36% | 2.63 |
| CL003954 | 8699 | 57% | 4.46 |
| CL022220 | 7645 | 52% | 3.92 |
| CL023130 | 7546 | 51% | 3.87 |
| CL023462 | 4134 | 29% | 2.12 |
| CL009131 | 4454.5 | 27% | 2.29 |
| CL017390 | 5763 | 41% | 2.96 |
| CL022448 | 5078 | 32% | 2.61 |
| CL054332 | 7062 | 49% | 3.62 |
| CL018994 | 4149.5 | 32% | 2.13 |
| CL019290 | 3195 | 20% | 1.64 |
| CL023374 | 6428 | 45% | 3.30 |
| CL023867 | 4152.5 | 27% | 2.13 |
| CL025404 | 6755 | 47% | 3.47 |
| CL024603 | 3905 | 25% | 2.00 |
| CL031124 | 4757 | 34% | 2.44 |
| CL024703 | 2983.5 | 14% | 1.53 |
| CL017492 | 2854.5 | 18% | 1.46 |
| CL020864 | 3349.5 | 16% | 1.72 |
| CL022957 | 2457.5 | 10% | 1.26 |
| CL011433 | 3473 | 19% | 1.78 |
| CL004467 | 12005 | 72% | 6.16 |
| CL014863 | 5260 | 38% | 2.70 |
| CL014538 | 4646 | 35% | 2.38 |
| CL012832 | 10350.5 | 60% | 5.31 |
| CL060680 | 4772 | 36% | 2.45 |
| CL023999 | 5096.5 | 35% | 2.62 |
| CL024381 | 4235 | 26% | 2.17 |
| CL064907 | 16407 | 83% | 8.42 |
| CL051676 | 16282 | 85% | 8.36 |
| CL021499 | 3384 | 25% | 1.74 |
| CL055152 | 2717 | 14% | 1.39 |
| CL059660 | 5964 | 41% | 3.06 |
| CL023980 | 8249 | 56% | 4.23 |
| CL006599 | 6164.5 | 43% | 3.16 |
| CL056103 | 14438.5 | 75% | 7.41 |
| CL054369 | 3343.5 | 23% | 1.72 |
| CL019599 | 7963.5 | 54% | 4.09 |
| CL033380 | 4460.5 | 31% | 2.29 |
| CL030257 | 5582.5 | 40% | 2.87 |
| CL018729 | 3293.5 | 24% | 1.69 |
| CL028535 | 15882.5 | 82% | 8.15 |
| CL051962 | 5676 | 41% | 2.91 |
| CL020834 | 7267.5 | 50% | 3.73 |
| CL032718 | 8789.5 | 56% | 4.51 |
| CL049229 | 9460 | 61% | 4.86 |
| CL055592 | 10129 | 67% | 5.20 |
| CL046358 | 24883 | 96% | 12.77 |
| CL055590 | 3191.5 | 21% | 1.64 |
| CL005380 | 3541 | 24% | 1.82 |
| CL058882 | 2949.5 | 16% | 1.51 |
| CL025179 | 15479.5 | 82% | 7.94 |
| CL002712 | 5253 | 38% | 2.70 |
| CL001010 | 5400.5 | 40% | 2.77 |
| CL021068 | 7000 | 49% | 3.59 |
| CL015420 | 5158 | 43% | 2.65 |
| CL012262 | 3226.5 | 20% | 1.66 |
| CL061025 | 4520.5 | 32% | 2.32 |
| CL028421 | 10011 | 60% | 5.14 |
| CL053546 | 3995 | 27% | 2.05 |
| CL023346 | 4892 | 34% | 2.51 |
| CL037193 | 4506.5 | 31% | 2.31 |
| CL024869 | 4202 | 30% | 2.16 |
| CL048882 | 2870 | 18% | 1.47 |
| CL012499 | 7219.5 | 50% | 3.71 |
| CL026196 | 4047 | 25% | 2.08 |
| CL055622 | 4048.5 | 26% | 2.08 |
| CL055698 | 4277 | 33% | 2.20 |
| CL002554 | 3914.5 | 28% | 2.01 |
| CL061290 | 5948 | 43% | 3.05 |
| CL027308 | 3222 | 22% | 1.65 |
| CL026640 | 5944 | 41% | 3.05 |
| CL050333 | 6444.5 | 47% | 3.31 |
| CL023599 | 4188.5 | 30% | 2.15 |
| CL039214 | 3883 | 24% | 1.99 |
| CL022632 | 3196.5 | 18% | 1.64 |
| CL002688 | 4560.5 | 30% | 2.34 |
| CL053625 | 4993.5 | 33% | 2.56 |
| CL053407 | 4572.5 | 30% | 2.35 |
| CL003906 | 9556 | 58% | 4.90 |
| CL054636 | 3639 | 21% | 1.87 |
| CL055641 | 5478 | 38% | 2.81 |
| CL055569 | 4894.5 | 33% | 2.51 |
| CL053606 | 2638.5 | 15% | 1.35 |
| CL055848 | 3454 | 22% | 1.77 |
| CL053591 | 3620 | 22% | 1.86 |
| CL020244 | 4012.5 | 27% | 2.06 |
| CL026537 | 5950 | 44% | 3.05 |
| CL055566 | 3804.5 | 23% | 1.95 |
| CL013450 | 4610.5 | 33% | 2.37 |
| CL055585 | 2922 | 13% | 1.50 |
| CL055832 | 3221.5 | 16% | 1.65 |
| CL023965 | 7179 | 50% | 3.68 |
| CL034006 | 4199 | 31% | 2.15 |
| CL007882 | 10423.5 | 68% | 5.35 |
| CL055855 | 2726.5 | 12% | 1.40 |
| CL002642 | 4927.5 | 35% | 2.53 |
| CL027783 | 4579 | 28% | 2.35 |
| CL053525 | 3869.5 | 26% | 1.99 |
| CL053808 | 5485.5 | 40% | 2.82 |
| CL023509 | 4544.5 | 31% | 2.33 |
| CL024014 | 15028 | 78% | 7.71 |
| CL055755 | 7146 | 50% | 3.67 |
| CL016950 | 5704 | 38% | 2.93 |
| CL035893 | 4688 | 33% | 2.41 |
| CL044071 | 6149.5 | 43% | 3.16 |
| CL037357 | 6783 | 48% | 3.48 |
| CL058779 | 4219 | 31% | 2.17 |

**Table title**

High-throughput screening of high-affinity monoclonal antibodies for lung cancer

**Table legend**

Table1. Antibody screening of mixed lung cancer cell lines (NCI-H520, NCI-H226, SK-MES-1, NCI-H2170, NCI-H69, NCI-H526) by flow cytometry.
