# Supplementary material for: Development of antibody drug conjugates targeting epithelial membrane protein 2-highly expressed lung cancer
Source: Cell Death Dis. 2025 Oct 31;16(1):771. doi: 10.1038/s41419-025-08125-7 (PMC12579237; doi:10.1038/s41419-025-08125-7)
Supplement: Supplementary file 2 — Supplemental Table 2 [file 41419_2025_8125_MOESM2_ESM.docx]

**Supplemental Table 2** SK-MES-1

| Antibody | MFI | Positive (%) | The ratio of antibody to IgG MFI |
| --- | --- | --- | --- |
| CL024015 | 14818 | 55% | 2.43 |
| CL027482 | 10835 | 29% | 1.78 |
| CL048862 | 8439.5 | 15% | 1.39 |
| CL029875 | 10130 | 29% | 1.66 |
| CL033251 | 33233.5 | 97% | 5.46 |
| CL030169 | 20542 | 81% | 3.37 |
| CL029847 | 26230 | 85% | 5.3 |
| CL025711 | 17604 | 69% | 2.89 |
| CL031337 | 20074 | 84% | 3.30 |
| CL031224 | 25904 | 89% | 4.25 |
| CL021989 | 17240.5 | 70% | 2.83 |
| CL029065 | 50720 | 99% | 8.33 |
| CL045144 | 18059 | 74% | 2.96 |
| CL034859 | 11804.5 | 37% | 1.94 |
| CL036063 | 11163 | 35% | 1.83 |
| CL003569 | 22652 | 84% | 3.72 |
| CL032725 | 9481.5 | 24% | 1.56 |
| CL032425 | 18487.5 | 70% | 3.03 |
| CL034563 | 43744 | 100% | 7.18 |
| CL035071 | 14185.5 | 52% | 2.33 |
| CL011665 | 23437 | 86% | 3.85 |
| CL028661 | 21887 | 90% | 3.59 |
| CL041708 | 15778.5 | 58% | 2.59 |
| CL007525 | 62792.5 | 98% | 10.31 |
| CL028841 | 17538.5 | 73% | 2.88 |
| CL038099 | 9424 | 25% | 1.55 |
| CL038316 | 9464.5 | 28% | 1.55 |
| CL012140 | 12472 | 41% | 2.05 |
| CL051358 | 19686.5 | 76% | 3.23 |
| CL051499 | 8657.5 | 21% | 1.42 |
| CL053171 | 7941 | 19% | 1.30 |
| CL033957 | 17076.5 | 64% | 2.80 |
| CL031384 | 15559.5 | 58% | 2.55 |
| CL028348 | 16912.5 | 66% | 2.78 |
| CL055882 | 9842 | 29% | 1.62 |
| CL025698 | 356557 | 100% | 58.53 |
| CL027015 | 9522.5 | 23% | 1.56 |
| CL031505 | 9669.5 | 28% | 1.59 |
| CL027153 | 19023.5 | 74% | 3.12 |
| CL016418 | 11044.5 | 36% | 1.81 |
| CL013501 | 16379 | 62% | 2.69 |
| CL055687 | 17328.5 | 67% | 2.84 |
| CL058789 | 14989.5 | 55% | 2.46 |
| CL009314 | 17067.5 | 68% | 2.80 |
| CL033308 | 11401 | 36% | 1.87 |
| CL019581 | 18074.5 | 74% | 2.97 |
| CL025051 | 51521 | 99% | 11.92 |
| CL026239 | 16366.5 | 79% | 3.79 |
| CL019772 | 23024 | 85% | 5.33 |
| CL037410 | 16799 | 79% | 3.89 |
| CL050737 | 32927.5 | 96% | 7.62 |
| CL023441 | 19325 | 90% | 4.47 |
| CL022327 | 12772 | 60% | 2.95 |
| CL027375 | 20458.5 | 90% | 4.73 |
| CL001026 | 13033 | 61% | 3.02 |
| CL030961 | 14605 | 70% | 3.38 |
| CL016308 | 158752 | 100% | 36.73 |
| CL035797 | 381756 | 100% | 88.32 |
| CL037030 | 11167 | 47% | 2.58 |
| CL032089 | 9611 | 40% | 2.22 |
| CL047885 | 15910 | 76% | 3.68 |
| CL033950 | 8076 | 33% | 1.87 |
| CL060313 | 15524 | 72% | 3.59 |
| CL025899 | 30721 | 95% | 7.11 |
| CL019384 | 25053 | 97% | 5.80 |
| CL013595 | 32922.5 | 98% | 7.62 |
| CL060117 | 19544 | 86% | 4.52 |
| CL010055 | 17814 | 81% | 4.12 |
| CL033749 | 75965.5 | 99% | 17.57 |
| CL031416 | 30238 | 98% | 7.00 |
| CL027213 | 109760 | 99% | 25.39 |
| CL008326 | 33122.5 | 97% | 7.66 |
| CL023772 | 27608 | 97% | 6.39 |
| CL004467 | 14968 | 67% | 3.46 |
| CL012832 | 18482 | 81% | 4.28 |
| CL064907 | 32577 | 98% | 7.54 |
| CL051676 | 14791.5 | 71% | 3.42 |
| CL056103 | 19995.5 | 81% | 4.63 |
| CL028535 | 35387 | 97% | 8.19 |
| CL055592 | 55275 | 98% | 12.79 |
| CL046358 | 102196 | 100% | 23.64 |
| CL025179 | 7061 | 24% | 1.63 |
| CL028421 | 21518 | 86% | 4.98 |
| CL007882 | 11667 | 50% | 2.70 |
| CL024014 | 12672.5 | 54% | 2.93 |

NCI-H226

| CL024015 | 26136.5 | 33% | 2.00 |
| --- | --- | --- | --- |
| CL027482 | 22334.5 | 26% | 1.71 |
| CL048862 | 17246.5 | 14% | 1.32 |
| CL029875 | 19218 | 21% | 1.47 |
| CL033251 | 68180 | 89% | 5.23 |
| CL030169 | 37905 | 61% | 2.91 |
| CL029847 | 20680 | 79% | 6.6 |
| CL025711 | 26518 | 37% | 2.03 |
| CL031337 | 50711 | 80% | 3.89 |
| CL031224 | 48173 | 76% | 3.69 |
| CL021989 | 21185.5 | 27% | 1.62 |
| CL029065 | 100895 | 96% | 7.73 |
| CL045144 | 19105.5 | 13% | 1.46 |
| CL034859 | 21124 | 24% | 1.62 |
| CL036063 | 20820 | 29% | 1.60 |
| CL003569 | 22409.5 | 30% | 1.72 |
| CL032725 | 16662.5 | 16% | 1.28 |
| CL032425 | 37636.5 | 61% | 2.89 |
| CL034563 | 70001.5 | 95% | 5.37 |
| CL035071 | 39380.5 | 63% | 3.02 |
| CL011665 | 24518.5 | 35% | 1.88 |
| CL028661 | 24166 | 31% | 1.85 |
| CL041708 | 32064.5 | 50% | 2.46 |
| CL007525 | 83993 | 93% | 6.44 |
| CL028841 | 24476 | 27% | 1.88 |
| CL038099 | 17809 | 17% | 1.37 |
| CL038316 | 23623 | 31% | 1.81 |
| CL012140 | 21253 | 24% | 1.63 |
| CL051358 | 20664.5 | 23% | 1.58 |
| CL051499 | 16695.5 | 15% | 1.28 |
| CL053171 | 16623.5 | 21% | 1.27 |
| CL033957 | 28506.5 | 43% | 2.19 |
| CL031384 | 50894 | 74% | 3.90 |
| CL028348 | 29963.5 | 46% | 2.30 |
| CL055882 | 23291.5 | 30% | 1.79 |
| CL025698 | 981395 | 100% | 75.23 |
| CL027015 | 46727 | 67% | 3.58 |
| CL031505 | 18597 | 22% | 1.43 |
| CL027153 | 42048 | 68% | 3.22 |
| CL016418 | 21396 | 29% | 1.64 |
| CL013501 | 30725 | 47% | 2.36 |
| CL055687 | 21584 | 26% | 1.65 |
| CL058789 | 16542 | 13% | 1.27 |
| CL009314 | 34269.5 | 55% | 2.63 |
| CL033308 | 20909 | 25% | 1.60 |
| CL019581 | 23865 | 31% | 1.83 |
| CL025051 | 35230.5 | 86% | 3.18 |
| CL026239 | 19135 | 37% | 1.73 |
| CL019772 | 18730 | 38% | 1.69 |
| CL037410 | 18403 | 34% | 1.66 |
| CL050737 | 32517 | 82% | 2.93 |
| CL023441 | 18759.5 | 0.3747 | 1.69 |
| CL022327 | 13513.5 | 0.116 | 1.22 |
| CL027375 | 19301.5 | 0.397 | 1.74 |
| CL001026 | 17169 | 0.291 | 1.55 |
| CL030961 | 17603 | 0.3065 | 1.59 |
| CL016308 | 44517.5 | 0.9565 | 4.02 |
| CL035797 | 371046 | 0.999 | 33.48 |
| CL037030 | 16312.5 | 26% | 1.47 |
| CL032089 | 21076.5 | 47% | 1.90 |
| CL047885 | 26358 | 68% | 2.38 |
| CL033950 | 23566.5 | 57% | 2.13 |
| CL060313 | 19857 | 42% | 1.79 |
| CL025899 | 31364.5 | 77% | 2.83 |
| CL019384 | 19834 | 40% | 1.79 |
| CL013595 | 24145.5 | 62% | 2.18 |
| CL060117 | 17507 | 32% | 1.58 |
| CL010055 | 18895.5 | 37% | 1.70 |
| CL033749 | 36150 | 86% | 3.26 |
| CL031416 | 21566.5 | 50% | 1.95 |
| CL027213 | 68386.5 | 99% | 6.17 |
| CL008326 | 36859 | 86% | 3.33 |
| CL023772 | 28148.5 | 74% | 2.54 |
| CL004467 | 15853.5 | 26% | 1.43 |
| CL012832 | 18428 | 37% | 1.66 |
| CL064907 | 33139 | 86% | 2.99 |
| CL051676 | 20362.5 | 43% | 1.84 |
| CL056103 | 19003 | 39% | 1.71 |
| CL028535 | 20813 | 48% | 1.88 |
| CL055592 | 39298 | 90% | 3.55 |
| CL046358 | 114753 | 100% | 10.35 |
| CL025179 | 12137 | 9% | 1.10 |
| CL028421 | 17918.5 | 34% | 1.62 |
| CL007882 | 18108 | 31% | 1.63 |
| CL024014 | 13890.5 | 12% | 1.25 |

NCI-H2170

| CL024015 | 34431.5 | 96% | 7.81 |
| --- | --- | --- | --- |
| CL027482 | 8045 | 46% | 1.83 |
| CL048862 | 6269.5 | 25% | 1.42 |
| CL029875 | 7354 | 42% | 1.67 |
| CL033251 | 84340 | 100% | 19.14 |
| CL030169 | 80023.5 | 100% | 18.16 |
| CL029847 | 13160 | 57% | 2.99 |
| CL025711 | 17297.5 | 92% | 3.92 |
| CL031337 | 15355 | 90% | 3.48 |
| CL031224 | 9326 | 57% | 2.12 |
| CL021989 | 7568 | 41% | 1.72 |
| CL029065 | 32051.5 | 100% | 7.27 |
| CL045144 | 13116.5 | 86% | 2.98 |
| CL034859 | 9239 | 56% | 2.10 |
| CL036063 | 8902 | 53% | 2.02 |
| CL003569 | 9161.5 | 55% | 2.08 |
| CL032725 | 10695 | 68% | 2.43 |
| CL032425 | 11846.5 | 72% | 2.69 |
| CL034563 | 34840 | 100% | 7.90 |
| CL035071 | 18579.5 | 88% | 4.22 |
| CL011665 | 8518.5 | 50% | 1.93 |
| CL028661 | 8368.5 | 49% | 1.90 |
| CL041708 | 13121.5 | 72% | 2.98 |
| CL007525 | 153956 | 100% | 34.93 |
| CL028841 | 13086 | 84% | 2.97 |
| CL038099 | 8374.5 | 49% | 1.90 |
| CL038316 | 7248 | 38% | 1.64 |
| CL012140 | 7133 | 39% | 1.62 |
| CL051358 | 8647 | 52% | 1.96 |
| CL051499 | 7450.5 | 40% | 1.69 |
| CL053171 | 5561 | 24% | 1.26 |
| CL033957 | 8067 | 46% | 1.83 |
| CL031384 | 11011.5 | 67% | 2.50 |
| CL028348 | 9659 | 60% | 2.19 |
| CL055882 | 6866.5 | 37% | 1.56 |
| CL025698 | 20917.5 | 95% | 4.75 |
| CL027015 | 6384 | 32% | 1.45 |
| CL031505 | 6235 | 29% | 1.41 |
| CL027153 | 19599.5 | 97% | 4.45 |
| CL016418 | 10749 | 63% | 2.44 |
| CL013501 | 13652 | 72% | 3.10 |
| CL055687 | 15776 | 79% | 3.58 |
| CL058789 | 18286 | 88% | 4.15 |
| CL009314 | 31808.5 | 99% | 7.22 |
| CL033308 | 7281.5 | 38% | 1.65 |
| CL019581 | 7703.5 | 43% | 1.75 |
| CL025051 | 19418.5 | 90% | 5.59 |
| CL026239 | 12715 | 80% | 3.66 |
| CL019772 | 8991.5 | 70% | 2.59 |
| CL037410 | 7583.5 | 63% | 2.18 |
| CL050737 | 15392 | 94% | 4.43 |
| CL023441 | 8551.5 | 0.7224 | 2.46 |
| CL022327 | 6092.5 | 0.436 | 1.75 |
| CL027375 | 10232.5 | 0.872 | 2.94 |
| CL001026 | 8822 | 0.7688 | 2.54 |
| CL030961 | 8809.5 | 0.7486 | 2.53 |
| CL016308 | 41735.5 | 0.9971 | 12.01 |
| CL035797 | 401874 | 0.9986 | 115.61 |
| CL037030 | 6119 | 44% | 1.76 |
| CL032089 | 6309.5 | 46% | 1.82 |
| CL047885 | 7809.5 | 66% | 2.25 |
| CL033950 | 23610 | 89% | 6.79 |
| CL060313 | 6142 | 44% | 1.77 |
| CL025899 | 11443 | 85% | 3.29 |
| CL019384 | 15141 | 97% | 4.36 |
| CL013595 | 9362.5 | 77% | 2.69 |
| CL060117 | 7622 | 64% | 2.19 |
| CL010055 | 65361 | 91% | 18.80 |
| CL033749 | 21817.5 | 96% | 6.28 |
| CL031416 | 10322.5 | 82% | 2.97 |
| CL027213 | 36614.5 | 100% | 10.53 |
| CL008326 | 10541 | 88% | 3.03 |
| CL023772 | 10521 | 88% | 3.03 |
| CL004467 | 6377.5 | 48% | 1.83 |
| CL012832 | 5475.5 | 33% | 1.58 |
| CL064907 | 15712.5 | 98% | 4.52 |
| CL051676 | 7436 | 62% | 2.14 |
| CL056103 | 7266.5 | 59% | 2.09 |
| CL028535 | 10128 | 83% | 2.91 |
| CL055592 | 4965 | 24% | 1.43 |
| CL046358 | 91418 | 100% | 26.30 |
| CL025179 | 3932 | 12% | 1.13 |
| CL028421 | 7021 | 56% | 2.02 |
| CL007882 | 4996 | 25% | 1.44 |
| CL024014 | 3957 | 11% | 1.14 |

NCI-H520

| CL024015 | 8013 | 93% | 5.09 |
| --- | --- | --- | --- |
| CL027482 | 4668.5 | 72% | 2.97 |
| CL048862 | 1996.5 | 19% | 1.27 |
| CL029875 | 3532 | 56% | 2.25 |
| CL033251 | 8323.5 | 95% | 5.29 |
| CL030169 | 16444 | 99% | 10.45 |
| CL029847 | 3690 | 13% | 1 |
| CL025711 | 8200 | 85% | 5.21 |
| CL031337 | 53250 | 100% | 33.85 |
| CL031224 | 10220.5 | 94% | 6.50 |
| CL021989 | 3577.5 | 60% | 2.27 |
| CL029065 | 96153.5 | 100% | 61.13 |
| CL045144 | 5950 | 93% | 3.78 |
| CL034859 | 8937 | 90% | 5.68 |
| CL036063 | 2403 | 35% | 1.53 |
| CL003569 | 3007.5 | 48% | 1.91 |
| CL032725 | 2870.5 | 44% | 1.82 |
| CL032425 | 5553.5 | 78% | 3.53 |
| CL034563 | 8690.5 | 98% | 5.52 |
| CL035071 | 11339 | 93% | 7.21 |
| CL011665 | 3733 | 59% | 2.37 |
| CL028661 | 3706 | 63% | 2.36 |
| CL041708 | 7835 | 84% | 4.98 |
| CL007525 | 44223.5 | 100% | 28.11 |
| CL028841 | 18859 | 99% | 11.99 |
| CL038099 | 2662 | 38% | 1.69 |
| CL038316 | 3182.5 | 52% | 2.02 |
| CL012140 | 8781.5 | 96% | 5.58 |
| CL051358 | 62872 | 100% | 39.97 |
| CL051499 | 2245 | 27% | 1.43 |
| CL053171 | 2070 | 26% | 1.32 |
| CL033957 | 5449 | 82% | 3.46 |
| CL031384 | 5566 | 77% | 3.54 |
| CL028348 | 5942.5 | 88% | 3.78 |
| CL055882 | 3714 | 59% | 2.36 |
| CL025698 | 6184.5 | 90% | 3.93 |
| CL027015 | 649343 | 100% | 412.81 |
| CL031505 | 3654 | 57% | 2.32 |
| CL027153 | 9481.5 | 95% | 6.03 |
| CL016418 | 4766 | 68% | 3.03 |
| CL013501 | 9691.5 | 90% | 6.16 |
| CL055687 | 7527 | 88% | 4.79 |
| CL058789 | 11783.5 | 91% | 7.49 |
| CL009314 | 9490 | 98% | 6.03 |
| CL033308 | 4750 | 72% | 3.02 |
| CL019581 | 19138.5 | 96% | 12.17 |
| CL025051 | 10449 | 96% | 8.58 |
| CL026239 | 7368.5 | 83% | 6.05 |
| CL019772 | 6302 | 75% | 5.17 |
| CL037410 | 3725 | 57% | 3.06 |
| CL050737 | 6024.5 | 81% | 4.95 |
| CL023441 | 3997 | 61% | 3.28 |
| CL022327 | 4818.5 | 73% | 3.96 |
| CL027375 | 3107.5 | 47% | 2.55 |
| CL001026 | 5539.5 | 71% | 4.55 |
| CL030961 | 5124 | 67% | 4.21 |
| CL016308 | 10173.5 | 94% | 8.35 |
| CL035797 | 29274.5 | 100% | 24.03 |
| CL037030 | 2774.5 | 40% | 2.28 |
| CL032089 | 2971 | 43% | 2.44 |
| CL047885 | 3842.5 | 58% | 3.15 |
| CL033950 | 2224 | 31% | 1.83 |
| CL060313 | 4051 | 59% | 3.33 |
| CL025899 | 4527.5 | 62% | 3.72 |
| CL019384 | 6040 | 78% | 4.96 |
| CL013595 | 2887 | 44% | 2.37 |
| CL060117 | 5393 | 79% | 4.43 |
| CL010055 | 11698.5 | 95% | 9.60 |
| CL033749 | 8356 | 85% | 6.86 |
| CL031416 | 3201 | 48% | 2.63 |
| CL027213 | 12254.5 | 95% | 10.06 |
| CL008326 | 6322.5 | 82% | 5.19 |
| CL023772 | 4330.5 | 65% | 3.56 |
| CL004467 | 4301.5 | 61% | 3.53 |
| CL012832 | 2009 | 25% | 1.65 |
| CL064907 | 6290.5 | 82% | 5.16 |
| CL051676 | 3956.5 | 60% | 3.25 |
| CL056103 | 3613 | 55% | 2.97 |
| CL028535 | 5727.5 | 77% | 4.70 |
| CL055592 | 12003.5 | 95% | 9.86 |
| CL046358 | 38369 | 100% | 31.50 |
| CL025179 | 1549 | 14% | 1.27 |
| CL028421 | 3284 | 50% | 2.70 |
| CL007882 | 3114 | 45% | 2.56 |
| CL024014 | 1820.5 | 19% | 1.49 |

NCI-H69

| CL024015 | 32999 | 99% | 15.83 |
| --- | --- | --- | --- |
| CL027482 | 31791 | 98% | 15.25 |
| CL048862 | 6695 | 63% | 3.21 |
| CL029875 | 41871 | 98% | 20.08 |
| CL033251 | 71913.5 | 100% | 34.49 |
| CL030169 | 37795 | 98% | 18.13 |
| CL029847 | 7970 | 47% | 2 |
| CL025711 | 119482 | 100% | 57.31 |
| CL031337 | 13552.5 | 95% | 6.50 |
| CL031224 | 14434 | 90% | 6.92 |
| CL021989 | 14867 | 93% | 7.13 |
| CL029065 | 103590 | 100% | 49.68 |
| CL045144 | 8758 | 77% | 4.20 |
| CL034859 | 83302 | 100% | 39.95 |
| CL036063 | 10227 | 79% | 4.91 |
| CL003569 | 13526 | 92% | 6.49 |
| CL032725 | 13090 | 86% | 6.28 |
| CL032425 | 30058.5 | 97% | 14.42 |
| CL034563 | 40400 | 100% | 19.38 |
| CL035071 | 158483 | 100% | 76.01 |
| CL011665 | 17346 | 95% | 8.32 |
| CL028661 | 12114 | 90% | 5.81 |
| CL041708 | 64872.5 | 99% | 31.11 |
| CL007525 | 70716 | 99% | 33.92 |
| CL028841 | 23359 | 98% | 11.20 |
| CL038099 | 8106 | 73% | 3.89 |
| CL038316 | 11100.5 | 85% | 5.32 |
| CL012140 | 7278.5 | 69% | 3.49 |
| CL051358 | 33141 | 99% | 15.89 |
| CL051499 | 6219 | 56% | 2.98 |
| CL053171 | 5303 | 47% | 2.54 |
| CL033957 | 19366 | 95% | 9.29 |
| CL031384 | 39819 | 99% | 19.10 |
| CL028348 | 26700 | 97% | 12.81 |
| CL055882 | 8819 | 72% | 4.23 |
| CL025698 | 13940.5 | 95% | 6.69 |
| CL027015 | 13854.5 | 89% | 6.64 |
| CL031505 | 53323 | 99% | 25.57 |
| CL027153 | 33548.5 | 98% | 16.09 |
| CL016418 | 25921 | 96% | 12.43 |
| CL013501 | 44088.5 | 99% | 21.15 |
| CL055687 | 53825.5 | 99% | 25.82 |
| CL058789 | 42897 | 99% | 20.57 |
| CL009314 | 58210 | 100% | 27.92 |
| CL033308 | 20446.5 | 96% | 9.81 |
| CL019581 | 14865 | 94% | 7.13 |
| CL025051 | 17754.5 | 100% | 9.82 |
| CL026239 | 22721 | 100% | 12.57 |
| CL019772 | 26149 | 100% | 14.46 |
| CL037410 | 14269 | 99% | 7.89 |
| CL050737 | 20558 | 100% | 11.37 |
| CL023441 | 11321 | 99% | 6.26 |
| CL022327 | 5292 | 85% | 2.93 |
| CL027375 | 11396.5 | 98% | 6.30 |
| CL001026 | 7175.5 | 93% | 3.97 |
| CL030961 | 9067 | 97% | 5.01 |
| CL016308 | 20295 | 100% | 11.23 |
| CL035797 | 143142 | 100% | 79.17 |
| CL037030 | 7755 | 96% | 4.29 |
| CL032089 | 8568 | 97% | 4.74 |
| CL047885 | 16880 | 99% | 9.34 |
| CL033950 | 11576.5 | 99% | 6.40 |
| CL060313 | 10322.5 | 97% | 5.71 |
| CL025899 | 8916 | 94% | 4.93 |
| CL019384 | 12644 | 99% | 6.99 |
| CL013595 | 8967 | 96% | 4.96 |
| CL060117 | 10338.5 | 99% | 5.72 |
| CL010055 | 16846 | 99% | 9.32 |
| CL033749 | 27970 | 100% | 15.47 |
| CL031416 | 13805 | 99% | 7.64 |
| CL027213 | 51976 | 100% | 28.75 |
| CL008326 | 23280 | 100% | 12.88 |
| CL023772 | 10824 | 99% | 5.99 |
| CL004467 | 21960 | 100% | 12.15 |
| CL012832 | 8584 | 96% | 4.75 |
| CL064907 | 16657 | 100% | 9.21 |
| CL051676 | 7717 | 95% | 4.27 |
| CL056103 | 7319 | 96% | 4.05 |
| CL028535 | 30407 | 100% | 16.82 |
| CL055592 | 22921 | 100% | 12.68 |
| CL046358 | 60259 | 100% | 33.33 |
| CL025179 | 4133 | 70% | 2.29 |
| CL028421 | 10409.5 | 99% | 5.76 |
| CL007882 | 6712 | 94% | 3.71 |
| CL024014 | 4414.5 | 76% | 2.44 |

NCI-H526

| CL024015 | 37483 | 95% | 11.46 |
| --- | --- | --- | --- |
| CL027482 | 37120.5 | 96% | 11.35 |
| CL048862 | 8406 | 49% | 2.57 |
| CL029875 | 44018.5 | 94% | 13.46 |
| CL033251 | 89102.5 | 100% | 27.25 |
| CL030169 | 93997.5 | 99% | 28.75 |
| CL029847 | 15800 | 72% | 4.4 |
| CL025711 | 112076 | 99% | 34.27 |
| CL031337 | 263712 | 100% | 80.65 |
| CL031224 | 18407.5 | 85% | 5.63 |
| CL021989 | 21183.5 | 86% | 6.48 |
| CL029065 | 91475 | 99% | 27.97 |
| CL045144 | 10969.5 | 64% | 3.35 |
| CL034859 | 53592.5 | 98% | 16.39 |
| CL036063 | 20204.5 | 82% | 6.18 |
| CL003569 | 31946 | 98% | 9.77 |
| CL032725 | 12233.5 | 66% | 3.74 |
| CL032425 | 38456 | 94% | 11.76 |
| CL034563 | 19965 | 90% | 6.11 |
| CL035071 | 160030 | 99% | 48.94 |
| CL011665 | 33498.5 | 95% | 10.24 |
| CL028661 | 22880 | 86% | 7.00 |
| CL041708 | 162573 | 99% | 49.72 |
| CL007525 | 89269.5 | 99% | 27.30 |
| CL028841 | 39537.5 | 93% | 12.09 |
| CL038099 | 8730.5 | 52% | 2.67 |
| CL038316 | 13695.5 | 69% | 4.19 |
| CL012140 | 13656 | 75% | 4.18 |
| CL051358 | 6619 | 36% | 2.02 |
| CL051499 | 10608.5 | 62% | 3.24 |
| CL053171 | 15104.5 | 77% | 4.62 |
| CL033957 | 26326.5 | 87% | 8.05 |
| CL031384 | 31281 | 93% | 9.57 |
| CL028348 | 13721 | 78% | 4.20 |
| CL055882 | 14459.5 | 74% | 4.42 |
| CL025698 | 25233 | 95% | 7.72 |
| CL027015 | 5742 | 27% | 1.76 |
| CL031505 | 16927.5 | 80% | 5.18 |
| CL027153 | 52092 | 97% | 15.93 |
| CL016418 | 109091 | 98% | 33.36 |
| CL013501 | 138365 | 99% | 42.31 |
| CL055687 | 114521 | 98% | 35.02 |
| CL058789 | 81651 | 99% | 24.97 |
| CL009314 | 27310.5 | 97% | 8.35 |
| CL033308 | 41160.5 | 96% | 12.59 |
| CL019581 | 9453.5 | 56% | 2.89 |
| CL025051 | 18078.5 | 85% | 4.87 |
| CL026239 | 53636.5 | 98% | 14.46 |
| CL019772 | 157038 | 99% | 42.35 |
| CL037410 | 28111 | 89% | 7.58 |
| CL050737 | 72889.5 | 99% | 19.65 |
| CL023441 | 16435.5 | 77% | 4.43 |
| CL022327 | 12938 | 67% | 3.49 |
| CL027375 | 36596 | 95% | 9.87 |
| CL001026 | 46794.5 | 99% | 12.62 |
| CL030961 | 48585 | 99% | 13.10 |
| CL016308 | 48353 | 99% | 13.04 |
| CL035797 | 521186 | 100% | 140.54 |
| CL037030 | 17262.5 | 74% | 4.65 |
| CL032089 | 27613.5 | 91% | 7.45 |
| CL047885 | 48704 | 96% | 13.13 |
| CL033950 | 27697.5 | 86% | 7.47 |
| CL060313 | 22993 | 88% | 6.20 |
| CL025899 | 18274.5 | 82% | 4.93 |
| CL019384 | 45452 | 95% | 12.26 |
| CL013595 | 59740 | 99% | 16.11 |
| CL060117 | 22663.5 | 93% | 6.11 |
| CL010055 | 23305.5 | 86% | 6.28 |
| CL033749 | 105407 | 99% | 28.42 |
| CL031416 | 23172 | 88% | 6.25 |
| CL027213 | 66235.5 | 99% | 17.86 |
| CL008326 | 85004 | 98% | 22.92 |
| CL023772 | 36335 | 97% | 9.80 |
| CL004467 | 55680 | 96% | 15.01 |
| CL012832 | 16180.5 | 78% | 4.36 |
| CL064907 | 36237 | 95% | 9.77 |
| CL051676 | 12395.5 | 64% | 3.34 |
| CL056103 | 20159 | 80% | 5.44 |
| CL028535 | 47025.5 | 96% | 12.68 |
| CL055592 | 18660.5 | 85% | 5.03 |
| CL046358 | 85590.5 | 99% | 23.08 |
| CL025179 | 7605.5 | 34% | 2.05 |
| CL028421 | 48143.5 | 95% | 12.98 |
| CL007882 | 28706 | 96% | 7.74 |
| CL024014 | 8533.5 | 38% | 2.30 |

**Table title**

High-throughput re-screening of high-affinity monoclonal antibodies for lung cancer

**Table legend**

Table2. Repeated antibody screening of single cell lines in lung cancer (SK-MES-1,NCI-H226,NCI-H2170,NCI-H520,NCI-H69,NCI-H526) by flow cytometry.
