# Supplementary material for: Development of antibody drug conjugates targeting epithelial membrane protein 2-highly expressed lung cancer
Source: Cell Death Dis. 2025 Oct 31;16(1):771. doi: 10.1038/s41419-025-08125-7 (PMC12579237; doi:10.1038/s41419-025-08125-7)
Supplement: Supplementary file 3 — Supplemental Material [file 41419_2025_8125_MOESM3_ESM.docx]

**Supplemental Figure and Table legends**
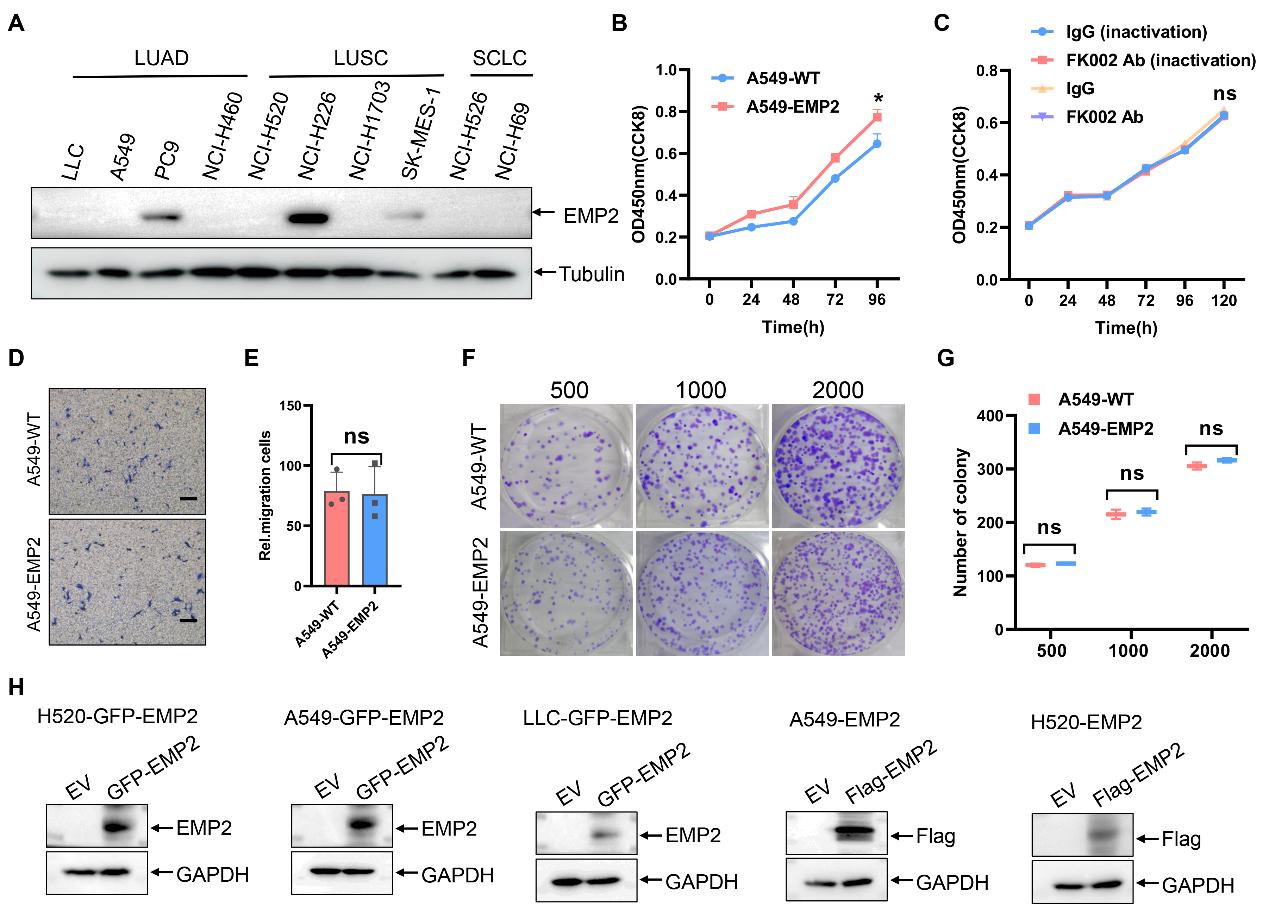


**Supplemental Figure 1. The target protein of FK002-exatecan has a limited effect on lung cancer cells**

(A) Immunoblot of anti-EMP2 from lung cancer cells (LLC, A549, PC9, NCI-H460, NCI-H520, NCI-H226, NCI-H1703, SK-MES-1, NCI-H526 and NCI-H69).

(B) Cell counting assay of wild type and EMP2-overexpressing A549 cells.

(C) Cell counting assay of A549 cells incubated with IgG (inactivation), FK002 Ab (inactivation), IgG, or FK002 Ab for the indicated times.

(D-E) Cell migration assay of wild type and EMP2-overexpressing A549 cells. Representative images were shown (Figure D). Quantitative data are shown in Figure E.

(F-G) Colony formation assay of wild type and EMP2-overexpressing A549 cells. Representative images were shown (Figure F). Quantitative data are shown in Figure G.

(H) Immunoblot of anti-EMP2 or anti-Flag from EMP2-overexpressing lung cancer cells (H520-GFP-EMP2, A549-GFP-EMP2, LLC-GFP-EMP2, A549-EMP2 and H520-EMP2). Data are expressed as mean ± SD of n = 3 independent experiments (B–G). ns, not significant; *p < 0.05.


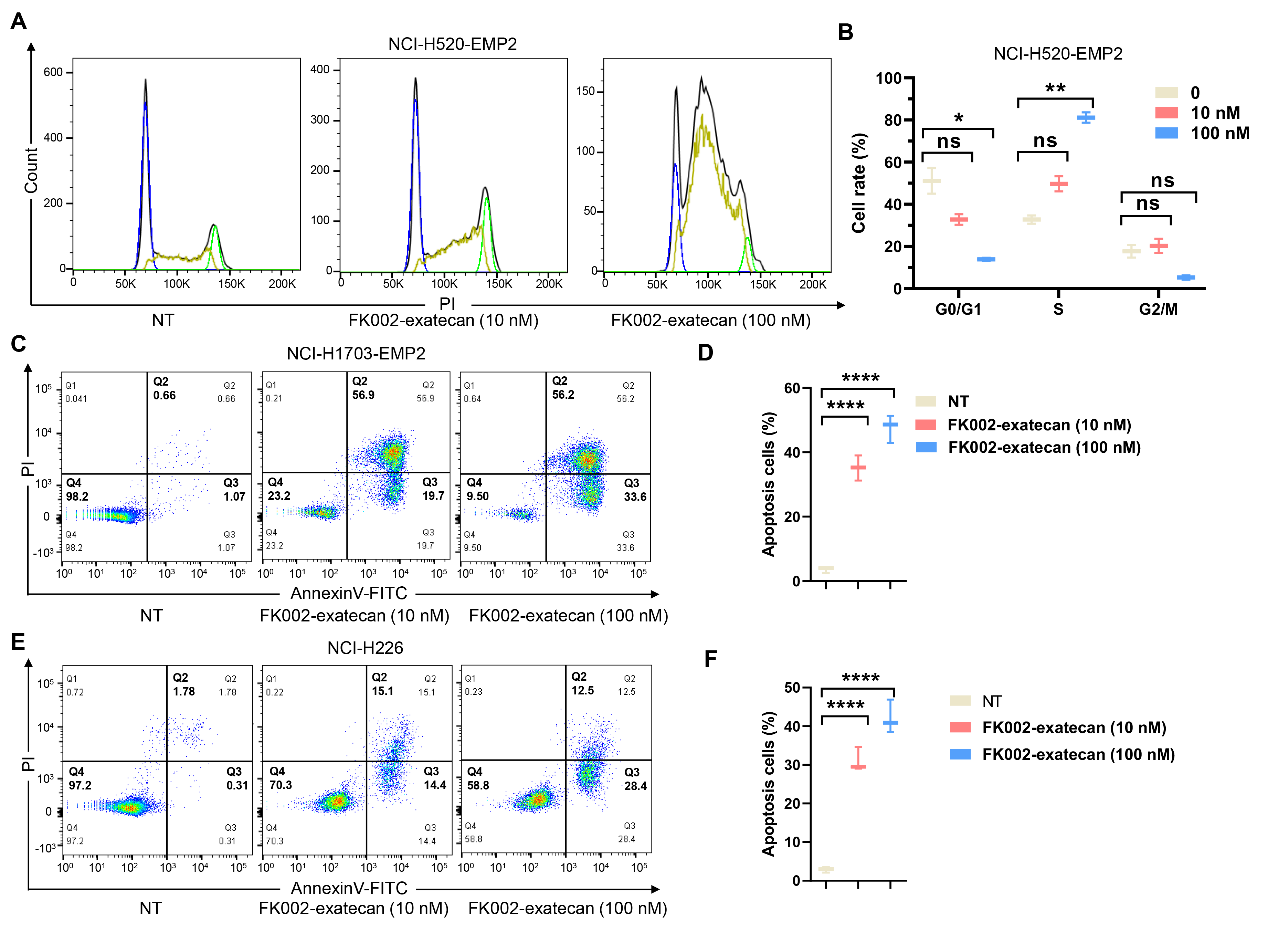


**Supplemental Figure 2.** **Induction of cell cycle arrest and apoptosis by FK002-exatecan in vitro**

(A-B) Cell cycle kinetic analysis of EMP2-overexpressing NCI-H520 cells treated with FK002-exatecan (0, 10, 100 nM) for 12 h (Figure A). Quantitative data are shown in Figure B.

(C-F) Flow cytometry of NCI-H1703-EMP2 and NCI-H226 cells treated with FK002-exatecan (0, 10, 100 nM) for 48 h. Quantitative data are shown in Figures D and F. Data are expressed as mean ± SD of n = 3 independent experiments (A–F). ns, not significant; *p < 0.05, **p < 0.01, ****p < 0.0001.


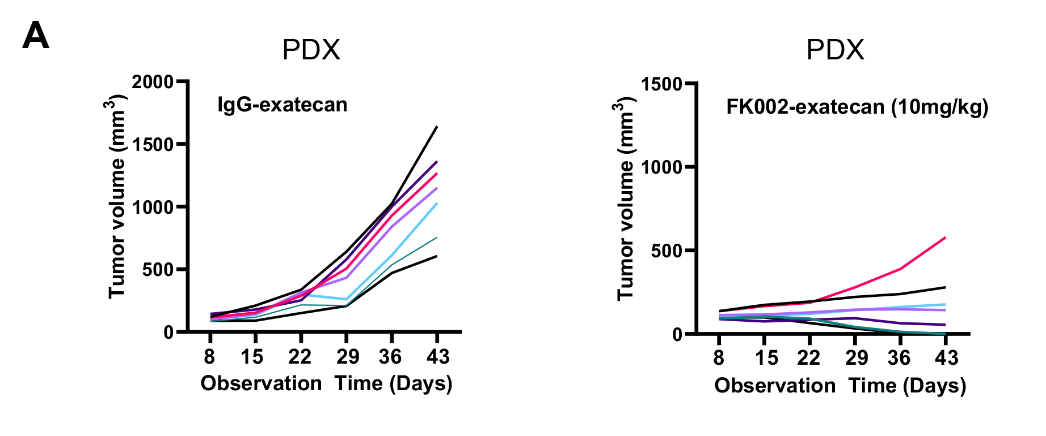


**Supplemental Figure 3.** **Antitumor activity of FK002-exatecan on patient-derived xenografts**

(A) Mice with NSCLC patient-derived xenograft tumors were treated with a single dose of 10 mg/kg FK002-exatecan or IgG-exatecan once per week for the indicated times. Tumor growth curves are displayed in Figure A.


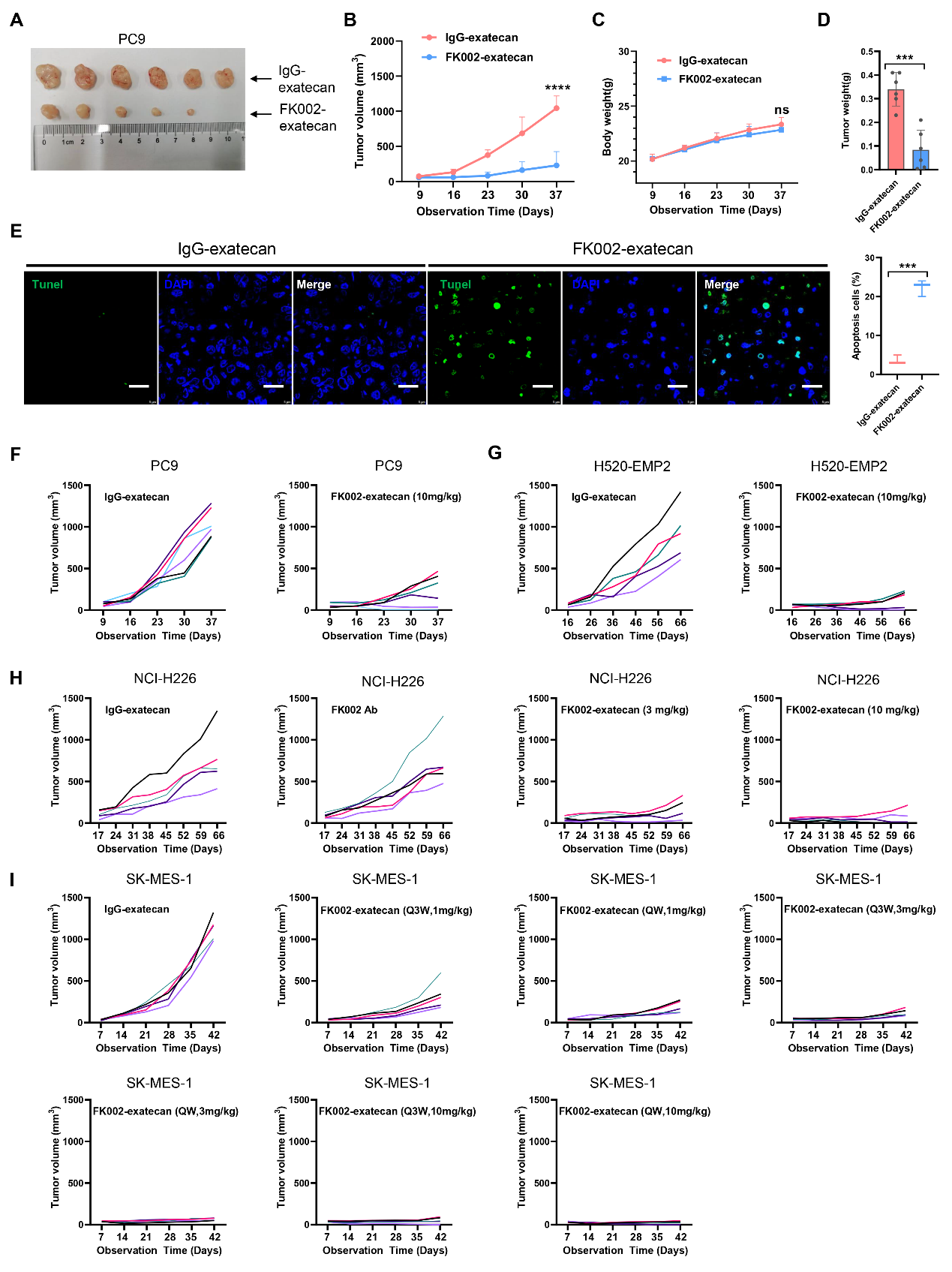


**Supplemental Figure 4. Antitumor activity of FK002-exatecan on xenograft tumors**

(A-D) Mice with PC9 xenograft tumors were treated with a single dose of 10 mg/kg FK002-exatecan or IgG-exatecan once per week for the indicated times. Tumor size (A), Tumor growth curve (B), Body weight (C) and Tumor weight (D) of tumors from xenograft models were displayed. Quantitative data are presented as means ± SD of six mice. Independent Student’s t-test. ns, not significant; ***P < 0.001; ****p < 0.0001.

(E) Tunel staining and quantitative data of tumor tissues from xenograft models are shown in E. Scale bars, 100 μm. Independent Student’s t-test. ***P < 0.001.

(F-I) Tumor growth curves from lung cancer cell lines (PC9, H520-EMP2, NCI-H226, and SK-MES-1) derived xenograft tumors were displayed.

**Supplemental table legends**

Table 1. Antibody screening of mixed lung cancer cell lines (NCI-H520, NCI-H226, SK-MES-1, NCI-H2170, NCI-H69, NCI-H526) by flow cytometry.

Table 2. Repeated antibody screening of single cell lines in lung cancer (SK-MES-1,NCI-H226,NCI-H2170,NCI-H520,NCI-H69,NCI-H526) by flow cytometry.
