# Supplementary figures and images for: Development of antibody drug conjugates targeting epithelial membrane protein 2-highly expressed lung cancer
Source: Cell Death Dis. 2025 Oct 31;16(1):771. doi: 10.1038/s41419-025-08125-7 (PMC12579237; doi:10.1038/s41419-025-08125-7)

Figure 2F

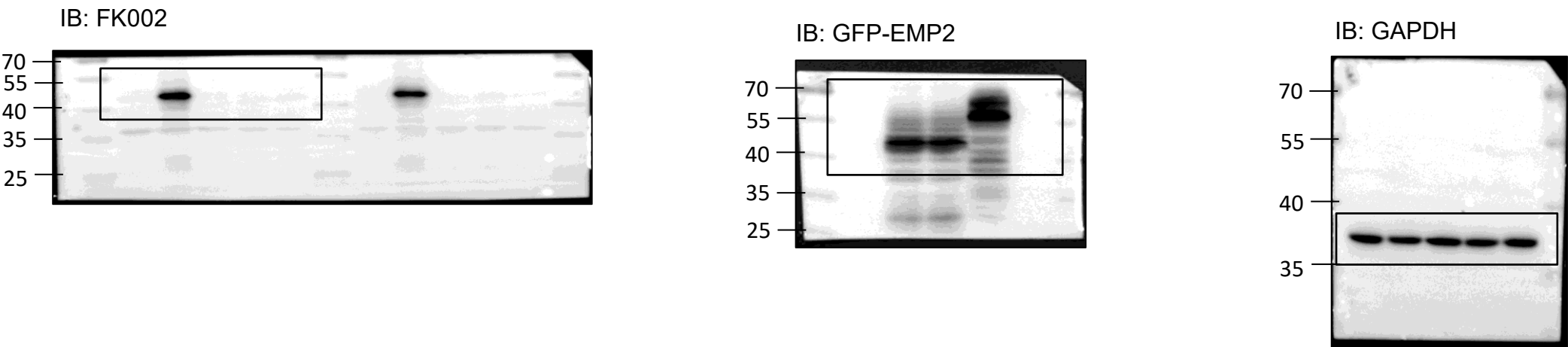

Figure 2I

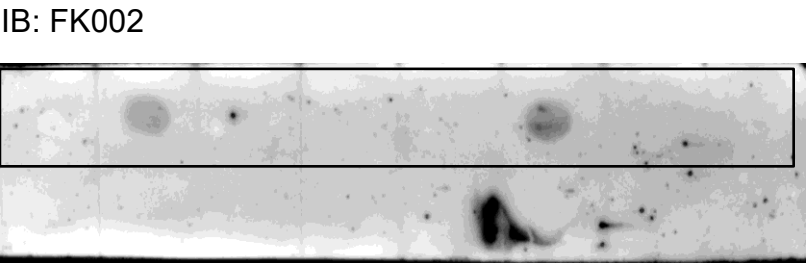

Figure S1A

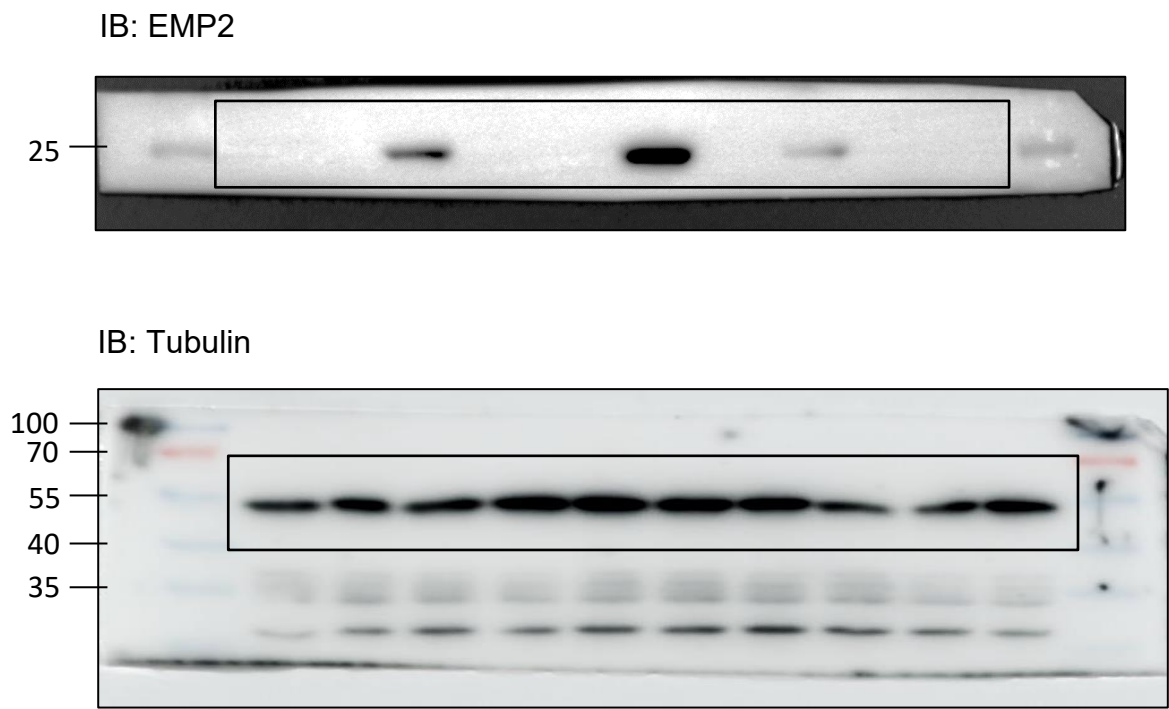

Figure S1H

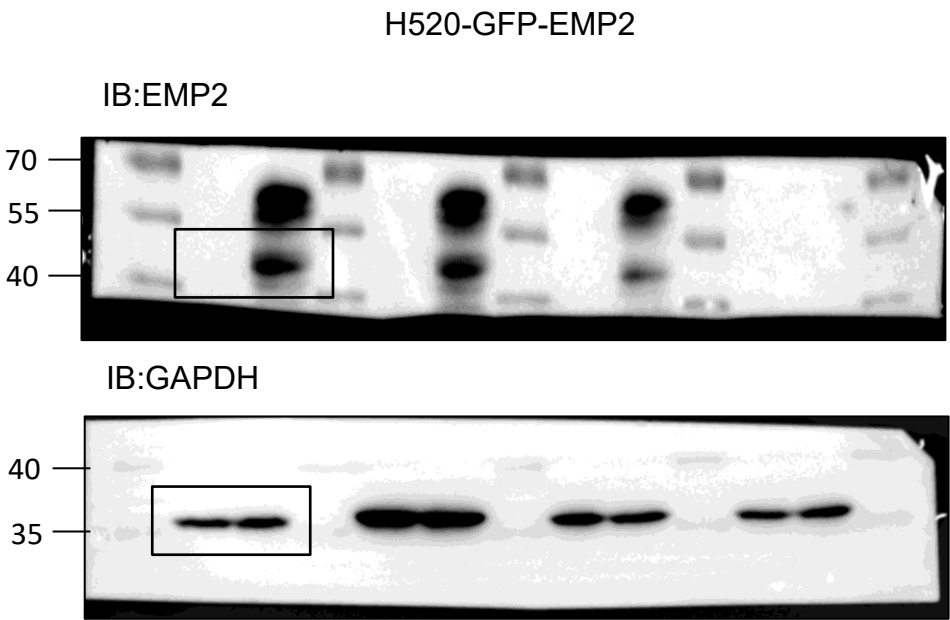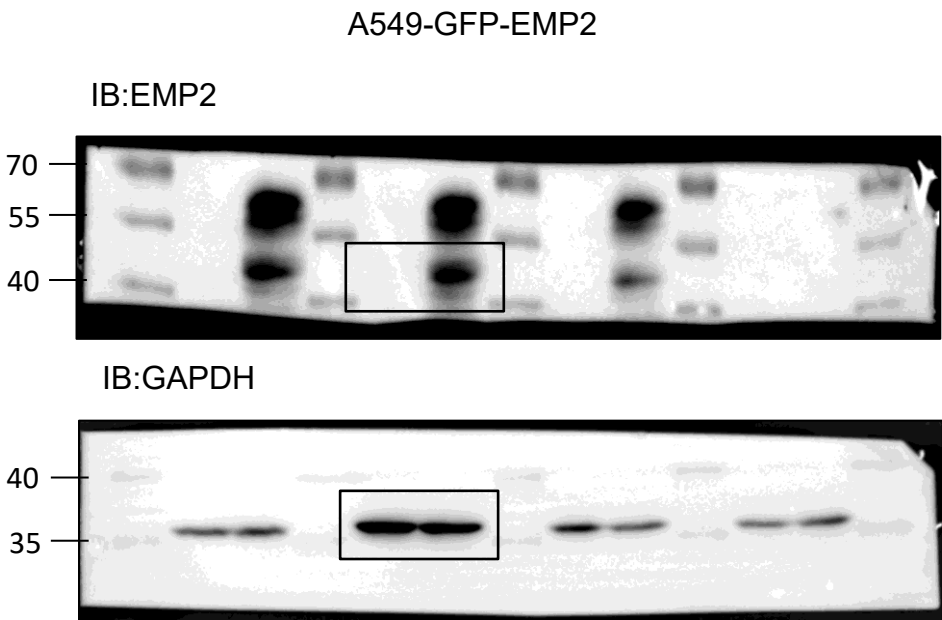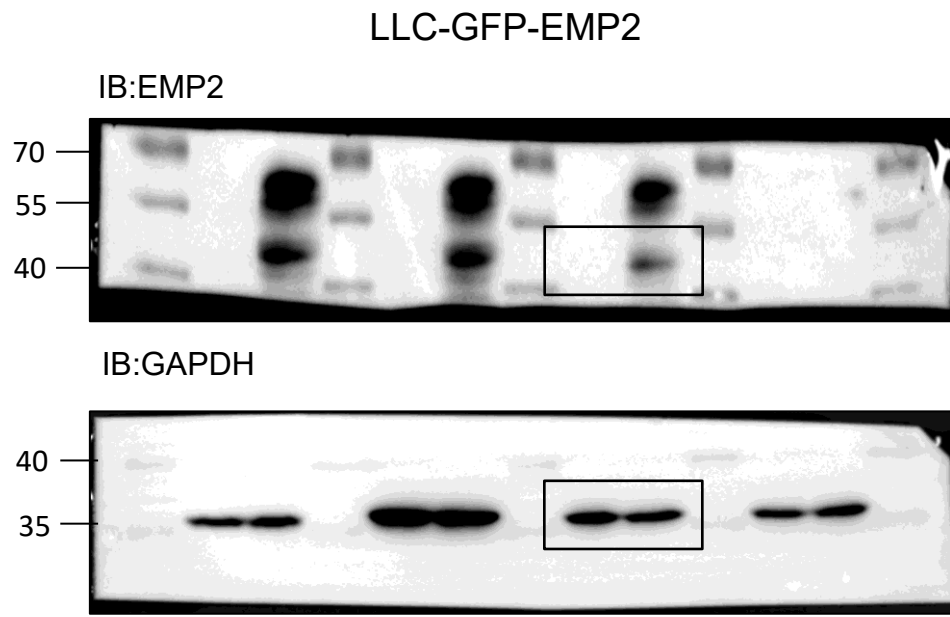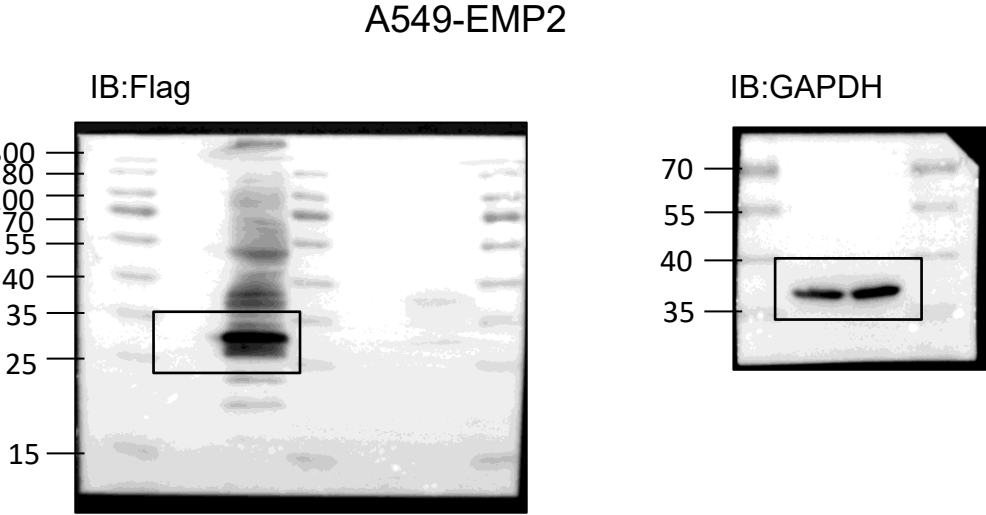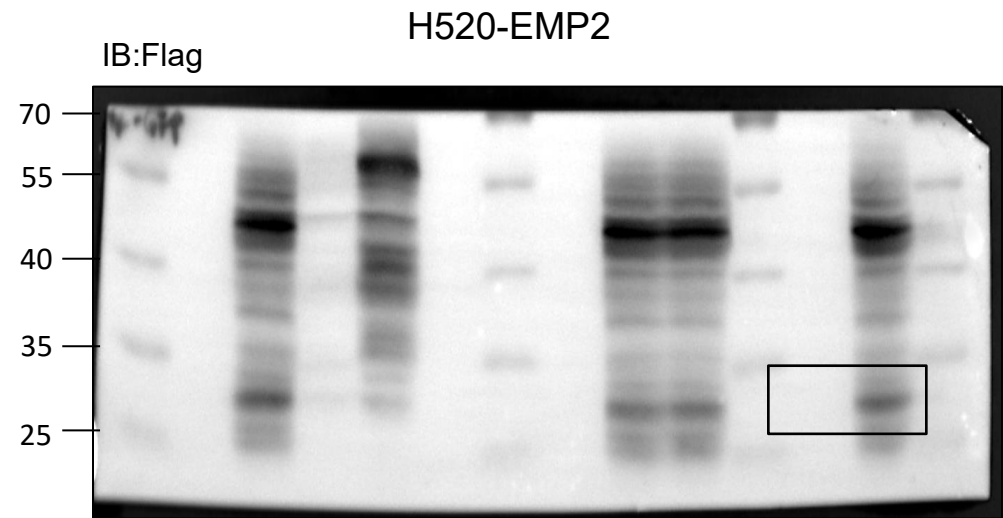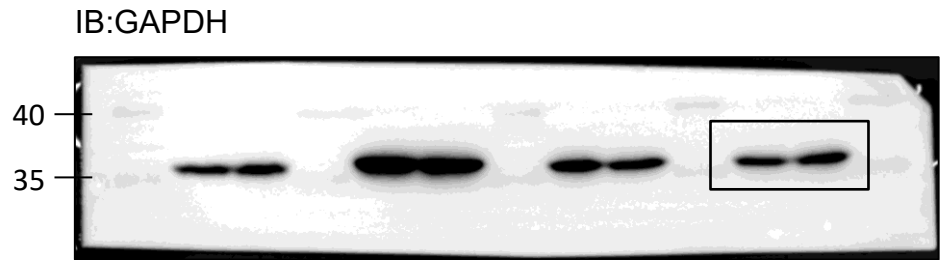

Supplement: Supplementary file 4 — original data [file 41419_2025_8125_MOESM4_ESM.pdf]
